# Supplementary material for: The importance of choosing appropriate methods for assessing wild food plant knowledge and use: A case study among the Baka in Cameroon
Source: PLoS One. 2021 Feb 18;16(2):e0247108. doi: 10.1371/journal.pone.0247108 (PMC7891729; doi:10.1371/journal.pone.0247108)
Supplement: S1 File — (DOCX) [file pone.0247108.s002.docx]

**S1 File.**

**EX-SITU INTERVIEWS**

FREE LISTING WILD EDIBLE PLANTS - Protocol for data collection questionnaire:

The aim is to gather all the wild edible plants known by the informants. The following question should be asked: “*Which food from the forest, excluding game, honey and mushrooms, do you know?”*

DIETARY DIVERSITY - Protocol for data collection questionnaire:

The approach to measuring dietary diversity is a qualitative 24-hour recall of all the foods and drinks consumed by the respondent. The respondent is asked about all foods he/she consumed the previous day, inside and outside the home. The most effective way to elicit the information on dietary diversity is to allow the respondent to freely recall what was eaten the previous day. This can be done as described below:

- Ask the respondent to list all the foods (meals and snacks) eaten the previous day during the day and night. Start with the first food/drink consumed during the morning.
- When the respondent recalls the food, write down the corresponding foods in the list under the appropriate food group.
- Probe for snacks eaten between main meals. Probe for what respondents ate while in the forest, or at their agricultural fields, when visiting other households, or while doing their daily activities outside the home.
- For each food respondents list, ask whether it was wild or cultivated (crop), and mark this in the data collection table. Ask them whether the respondent obtained it from the market or by means of barter with the neighbors or other people.
- Make sure you include even small quantities of food eaten, especially if it regards wild edibles. With processed foods, however, for instance a dash of milk to just lighten the coffee may be considered too small an amount to count the milk in the milk products group. Include salt, sugar and oil, as there seem to be variations in the use of these within the community.

What did you eat the last 24 hours ? What did you eat as meals and snacks? What did you drink? *If the previous day was a celebration or feast, did you eat special foods or eat more or less than usual.”*

| **Name:** | | **Subject ID:** | | **Village** | | **Date:** |
| --- | --- | --- | --- | --- | --- | --- |
| **Food item consumed** | **Quantity** | | **Number of people eating from same meal** | | **Cultivated / Wild / Market / Barter** | |
|  |  | |  | |  | |
|  |  | |  | |  | |
|  |  | |  | |  | |
|  |  | |  | |  | |
|  |  | |  | |  | |
|  |  | |  | |  | |
|  |  | |  | |  | |
|  |  | |  | |  | |

INCOME FROM SALE - Protocol for data collection questionnaire:

To obtain data on all tangible items sold and bartered by adults informants during the **two weeks prior** to the interview (recall period = 14 days). The following question should be asked to each informant: **“***What have you sold, bartered, or traded in the past two weeks?”*

If a product was bartered (traded), then find out later the **total market price** (note: not village price!) of the product received (i.e. unit price x quantity received).

**Questionnaire:**

|  | |  | | | **Date:** | |  | |
| --- | --- | --- | --- | --- | --- | --- | --- | --- |
|  | | | | | | | | |
| **Subject ID** | **Item sold** | | **Quantity** | **Units** | | **Total Value*** | | **Sale** |
|  | If bartered, put item given | | Number |  | | if bartered: value of item received in place of reception | | cash=1; barter=0 |
|  |  | |  |  | |  | |  |
|  |  | |  |  | |  | |  |
|  |  | |  |  | |  | |  |
|  |  | |  |  | |  | |  |
|  |  | |  |  | |  | |  |
|  |  | |  |  | |  | |  |
|  |  | |  |  | |  | |  |
|  |  | |  |  | |  | |  |
|  |  | |  |  | |  | |  |

**IN SITU INTERVIEWS**

WALK-IN-THE-WOOD QUESTIONNAIRE

Protocol for data collection:

As soon as a wild plant is shown by one of the informants, then ask:

1. the local name of the plants, in Baka and Nzime or other languages known

2. which parts is consumed

3. how the edible part is prepared

4. When was the last time the informant ate this plant?

< 2 days ago

< 1 week ago

< 1 month ago

< 1 year ago

< 2 years ago

> 2 years ago

Never

5. Is there a part of this plant sold to middlemen or on the local market?

6. Is the wood logged by timber companies?

If known, record the tentative scientific name and family name, and the vegetation type.

Record signs of harvesting.
