# Supplementary material for: The importance of choosing appropriate methods for assessing wild food plant knowledge and use: A case study among the Baka in Cameroon
Source: PLoS One. 2021 Feb 18;16(2):e0247108. doi: 10.1371/journal.pone.0247108 (PMC7891729; doi:10.1371/journal.pone.0247108)
Supplement: Attachment — Submitted filename: PONE-D-20-37238_R1_reviewer.pdf [file pone.0247108.s004.pdf]

PLOS ONE

The importance of choosing appropriate methods for assessing wild food plant knowledge and use: a case study among the Baka in Cameroon.

--Manuscript Draft--

**Manuscript Number:**	PONE-D-20-37238R1
**Article Type:**	Research Article
**Full Title:**	The importance of choosing appropriate methods for assessing wild food plant knowledge and use: a case study among the Baka in Cameroon.
**Short Title:**	Choosing appropriate methods for assessing wild food plant knowledge.
**Corresponding Author:**	Sandrine Gallois<br>Leiden University: Universiteit Leiden<br>Leiden, NETHERLANDS
**Keywords:**	Ethnobotany, Hunter-gatherers, Freelisting, Dietary Recall, Walk-in-the-woods, Wild food plants.
**Abstract:**	In tropical rainforests, access to and availability of natural resources are vital for the dietary diversity and food security of forest-dwelling societies. In the Congo Basin, these are challenged by the increasing exploitation of forests for bushmeat, commercial hardwood, mining, and large-scale agriculture. In this context, a balanced approach is needed between the pressures from forest exploitation, non-timber forest product trade and the livelihood and dietary behavior of rural communities. While there is a general positive association between tree cover and dietary diversity, the complex biocultural interactions between tropical forest food resources and the communities they sustain are still understudied. This research focuses on the knowledge and use of wild food plants by the forest-dwelling Baka people in southeast Cameroon. By using two different sets of methods, namely ex-situ interviews and in-situ surveys, we collected ethnographic and ethnobotanical data in two Baka settlements and explored the diversity of wild edible plants known, the frequency of their consumption, and potential conflicts between local diet and commercial trade in forest resources. Within a single Baka population, we showed that the in-situ walk-in-the-woods method resulted in more detailed information on wild food plant knowledge and use frequency than the ex-situ methods of freelisting and dietary recalls. Our in-situ method yielded 91 wild edible species, much more than the ex-situ freelisting interviews (38 spp.) and dietary recalls (12 spp.). Our results suggest that studies that are based only on ex-situ interviews may underestimate the importance of wild food plants for local communities. We discuss the limitations and strengths of these different methods for capturing the diversity of wild food plant knowledge and uses and propose that future studies on wild food plant knowledge and use frequency should rely on a mixed approach that combines in -situ and ex-situ methods.
**Order of Authors:**	Sandrine Gallois
	Thomas Heger
	Amanda Georganna Henry
	Tinde van Andel
**Response to Reviewers:**	Anna FodorLeiden, 6 January, 2021<br>Academic Editor<br>PLOS ONE<br><br>Dear Dr. Fodor,<br>Thank you for allowing us to adapt our manuscript PONE-D-20-37238, entitled “The importance of choosing appropriate methods for assessing wild food plant knowledge and use: a case study among the Baka in Cameroon”. We have followed your suggestions and hope that the manuscript now fits the submission standards of PLoS ONE.<br>We have submitted a revised version of our manuscript labeled as “revised manuscript with track changes” and a clean copy (unmarked) labeled as “manuscript”, as well as the response to the Editor's comments, Dr Muhammad Ishtiaq, in the document

Powered by Editorial Manager® and ProduXion Manager® from Aries Systems Corporation

	"Response to Reviewer" with a point by point answers.<br><br>Below, we list each point made by the academic editor, and our respective answer.<br>Additional Editor Comments:<br><br>Paper needs followings changes/improvements and data provisions; in order to proceed it for further.<br>(1) needs to improve English language, there are many mistakes in the article.(letter of English experts reviewed as supporting needed.<br>Answer to comment #1: Our manuscript was checked by someone with ample experience in publishing scientific articles in the English language.<br><br>(2) Letter of Ethical and ISE and other institution permission letters are required.<br>Answer to comment #2: The International Society for Ethnobiology does not issue permission letters and does not have an ethnical board: it just published standards for researchers, to which we adhered. However, our research was carried out within the ERC project “HARVEST: Plant Foods in Human Evolution; Factors affecting the harvest of nutrients from the floral environment”. For this research project, our co-author Amanda Henry received approval from the Ethical Board of the Faculty of Medicine of the University of Leipzig, Germany. We have added this letter to our submitted files and listed the HARVEST project in the funding. Moreover, before we started our fieldwork, we received permission from the National Committee of Ethics for the Research of Human Health (CNERSH) of the Cameroonian government. We also added this letter to the submitted files.<br><br>(3) The references cited in text and reference section needs to be rechecked as it has many mistakes in it.<br>Answer to comment #3: We have checked all references in the text and reference list and adapted them to the style of PLoS ONE.<br><br>(4) Figure of study area needed.<br>Answer to comment #4: We included a map of the study area (Figure 1)<br><br>(5) Model of questionnaire needed.<br>Answer to comment #5: We included the model of our questionnaire in our Supporting Information (Supplementary file 1).<br><br>(6) Families names are not correct, recheck and resend table 1.<br>Answer to comment #6: Family and author names of the species are now listed in the Supporting Information (Supplementary Table 1). All scientific names were checked and updated suing Plants of the World Online.<br><br>(7) Provide ubiquitous herbarium numbers for all plants collected. it has two types of numbering.<br>Answer to comment #7: Herbarium numbers for all plants collected are listed in Supplementary Table 1, but there are two acronyms (WTH and SG), referring to the two main collectors: Willem Thomas Heger and Sandrine Gallois. We had to follow the Naturalis guidelines of specimen collection for this study.<br><br>(8) Figures are dim/blurred, send high density/good quality figures.<br>Answer to comment #8: We have now uploaded high quality figures following the guidelines of PLoS ONE.<br>On behalf of our co-authors, we hope that our manuscript is now suitable to be considered for review,<br>Yours sincerely,<br>Sandrine Gallois and Tinde van Andel
**Additional Information:**	
**Question**	**Response**
**Financial Disclosure**<br><br>Enter a financial disclosure statement that describes the sources of funding for the	This research has received funding from the European Research Council under the European Union’s Horizon 2020 research and innovation program; grant agreement number STG–677576 (“HARVEST”) awarded to AGH. The botanical fieldwork was funded by Naturalis Biodiversity Center (https://www.naturalis.nl/en)to TVA, the

work included in this submission. Review the submission guidelines for detailed requirements. View published research articles from *PLOS ONE* for specific examples.<br><br>This statement is required for submission and **will appear in the published article** if the submission is accepted. Please make sure it is accurate.<br><br>**Unfunded studies**<br>Enter: *The author(s) received no specific funding for this work.*<br><br>**Funded studies**<br>Enter a statement with the following details:<br>• Initials of the authors who received each award<br>• Grant numbers awarded to each author<br>• The full name of each funder<br>• URL of each funder website<br>• Did the sponsors or funders play any role in the study design, data collection and analysis, decision to publish, or preparation of the manuscript?<br>• **NO** - Include this sentence at the end of your statement: *The funders had no role in study design, data collection and analysis, decision to publish, or preparation of the manuscript.*<br>• **YES** - Specify the role(s) played.<br><br>* typeset	TreubMaatschappij and the Alberta Mennega Stichting (https://www.alberta-mennega-stichting.nl) to TH. None of the funders has played a role in the research design, data collection, analysis, decision to publish or preparation of the manuscript.
**Competing Interests**<br><br>Use the instructions below to enter a competing interest statement for this submission. On behalf of all authors, disclose any competing interests that could be perceived to bias this work—acknowledging all financial support and any other relevant financial or non-financial competing interests.<br><br>This statement **will appear in the published article** if the submission is accepted. Please make sure it is accurate. View published research articles from *PLOS ONE* for specific examples.	The authors have declared that no competing interests exist.

Powered by Editorial Manager® and ProduXion Manager® from Aries Systems Corporation

**NO authors have competing interests**<br><br>Enter: *The authors have declared that no competing interests exist.*<br><br>**Authors with competing interests**<br><br>Enter competing interest details beginning with this statement:<br><br>*I have read the journal's policy and the authors of this manuscript have the following competing interests: [insert competing interests here]*<br><br>* typeset	
**Ethics Statement**<br><br>Enter an ethics statement for this submission. This statement is required if the study involved:<br><br>• Human participants<br>• Human specimens or tissue<br>• Vertebrate animals or cephalopods<br>• Vertebrate embryos or tissues<br>• Field research<br><br>Write "N/A" if the submission does not require an ethics statement.<br><br>General guidance is provided below. Consult the submission guidelines for detailed instructions. **Make sure that all information entered here is included in the Methods section of the manuscript.**	This research was carried out within the ERC project "HARVEST: Plant Foods in Human Evolution; Factors affecting the harvest of nutrients from the floral environment". For this research project, Amanda G. Henry received approval from the Ethical Commission of the Faculty of Medicine of the University of Leipzig, Germany (196-16/ek). Moreover,<br>before we started our fieldwork, we received permission from the Ethical Committee from the Ministry of Health of Cameroon (n°2018/06/1049/CE/CNERSH/SP).<br>This study also adheres to the Code of Ethics of the International Society of Ethnobiology (2006), and the authors received oral and written Free Prior Informed Consent from all the participants before beginning data collection.

Powered by Editorial Manager® and ProduXion Manager® from Aries Systems Corporation

**Format for specific study types**<br><br>**Human Subject Research (involving human participants and/or tissue)**<br>• Give the name of the institutional review board or ethics committee that approved the study<br>• Include the approval number and/or a statement indicating approval of this research<br>• Indicate the form of consent obtained (written/oral) or the reason that consent was not obtained (e.g. the data were analyzed anonymously)<br><br>**Animal Research (involving vertebrate animals, embryos or tissues)**<br>• Provide the name of the Institutional Animal Care and Use Committee (IACUC) or other relevant ethics board that reviewed the study protocol, and indicate whether they approved this research or granted a formal waiver of ethical approval<br>• Include an approval number if one was obtained<br>• If the study involved *non-human primates*, add *additional details* about animal welfare and steps taken to ameliorate suffering<br>• If anesthesia, euthanasia, or any kind of animal sacrifice is part of the study, include briefly which substances and/or methods were applied<br><br>**Field Research**<br><br>Include the following details if this study involves the collection of plant, animal, or other materials from a natural setting:<br>• Field permit number<br>• Name of the institution or relevant body that granted permission	
**Data Availability**<br><br>Authors are required to make all data underlying the findings described fully available, without restriction, and from the time of publication. PLOS allows rare exceptions to address legal and ethical concerns. See the PLOS Data Policy and FAQ for detailed information.	Yes - all data are fully available without restriction

A Data Availability Statement describing where the data can be found is required at submission. Your answers to this question constitute the Data Availability Statement and **will be published in the article**, if accepted.<br><br>**Important:** Stating 'data available on request from the author' is not sufficient. If your data are only available upon request, select 'No' for the first question and explain your exceptional situation in the text box.<br><br>Do the authors confirm that all data underlying the findings described in their manuscript are fully available without restriction?	
**Describe where the data may be found in full sentences. If you are copying our sample text, replace any instances of XXX with the appropriate details.**<br><br>• If the data are **held or will be held in a public repository**, include URLs, accession numbers or DOIs. If this information will only be available after acceptance, indicate this by ticking the box below. For example: *All XXX files are available from the XXX database (accession number(s) XXX, XXX.*).<br>• If the data are all contained **within the manuscript and/or Supporting Information files**, enter the following: *All relevant data are within the manuscript and its Supporting Information files.*<br>• If neither of these applies but you are able to provide **details of access elsewhere**, with or without limitations, please do so. For example:<br><br>*Data cannot be shared publicly because of [XXX]. Data are available from the XXX Institutional Data Access / Ethics Committee (contact via XXX) for researchers who meet the criteria for access to confidential data.*<br><br>*The data underlying the results presented in the study are available from (include the name of the third party*	All relevant data are within the manuscript and its Supporting Information files (Supplementary Table 1).

and contact information or URL).<br>• This text is appropriate if the data are owned by a third party and authors do not have permission to share the data.<br><br>* typeset	
Additional data availability information:	Tick here if the URLs/accession numbers/DOIs will be available only after acceptance of the manuscript for publication so that we can ensure their inclusion before publication.

Powered by Editorial Manager® and ProduXion Manager® from Aries Systems Corporation

Manuscript Click here to access/download;Manuscript;Manuscript.docx

The importance of choosing appropriate methods for assessing wild food plant knowledge and use: a case study among the Baka in Cameroon.

Short title: Choosing appropriate methods for assessing wild food plant knowledge.

Sandrine Gallois[1*], Thomas Heger[2], Amanda G. Henry[1], and Tinde van Andel[2,3]

[1]Faculty of Archaeology, Leiden University, Leiden, the Netherlands
[2]Biosystematics Group, Wageningen University, Wageningen, the Netherlands
[3]Naturalis Biodiversity Center, Leiden, the Netherlands

*Corresponding Author: Sandrine Gallois
Faculty of Archaeology, Leiden University, Leiden, the Netherlands

Email: galloissandrine.ethnoeco@gmail.com

Abstract

In tropical rainforests, access to and availability of natural resources are vital for the dietary diversity and food security of forest-dwelling societies. In the Congo Basin, these are challenged by the increasing exploitation of forests for bushmeat, commercial hardwood, mining, and large-scale agriculture. In this context, a balanced approach is needed between the pressures from forest exploitation, non-timber forest product trade and the livelihood and dietary behavior of rural communities. While there is a general positive association between tree cover and dietary diversity, the complex biocultural interactions between tropical forest food resources and the communities they sustain are still understudied. This research focuses on the knowledge and use of wild food plants by the forest-dwelling Baka people in southeast Cameroon. By using two different sets of methods, namely *ex-situ* interviews and *in-situ* surveys, we collected ethnographic and ethnobotanical data in two Baka settlements and explored the diversity of wild edible plants known, the frequency of their consumption, and potential conflicts between local diet and commercial trade in forest resources. Within a single Baka population, we showed that the *in-situ* walk-in-the-woods method resulted in more detailed information on wild food plant knowledge and use frequency than the *ex-situ* methods of freelisting and dietary recalls. Our *in-situ* method yielded 91 wild edible species, much more than the *ex-situ* freelisting interviews (38 spp.) and dietary recalls (12 spp.). Our results suggest that studies that are based only on *ex-situ* interviews may underestimate the importance of wild food plants for local communities. We discuss the limitations and strengths of these different methods for capturing the diversity of wild food plant knowledge and uses and propose that future studies on wild food plant knowledge and use frequency should rely on a mixed approach that combines *in-situ* and *ex-situ* methods.

Introduction

In tropical rainforests, access to and availability of natural resources are vital for the dietary diversity and food security of forest-dwelling societies. The Congo Basin includes the largest tract of tropical rainforest in Africa. This highly biodiverse region is inhabited by more than 30 million people, including forest-dwelling communities that strongly depend on their natural environment for their survival [1]. A wide variety of wild edible plant species (WEP) has been reported for the Congo Basin, among Bantu-speaking farmers and especially among hunter-gatherers that infrequently practice agriculture [2-6]. Local people harvest these wild food plants mainly for subsistence use within the household, but a substantial number of species is locally traded, and a few find their way to global markets [7, 8]. The cash income earned with the trade in non-timber forest products (NTFPs), important for improving livelihoods, can empower local communities [9]. Wild edible plants also provide a safety net in times of agricultural shortage and are thus essential for food security [9]. However, forest-dwelling societies in the Congo Basin are challenged by a decrease in access to and availability of wild food resources due to the increasing exploitation of the forests for commercial hardwood, precious minerals, and space for oil palm and rubber plantations [10, 11]. As a result of large-scale agricultural encroachment, logging, mining and hunting activities, the ecosystems of this region are under severe pressure [10, 12]. A recent survey estimated that just 70% of the standing forests of the Congo Basin remain fully intact [11]. Pressures on local resources have led to a decrease in local people's access to the important wild plants and bushmeat, which contributes substantially to their diet and medicine [13, 14]. In Cameroon, for example, more than 60% of the top 23 timber species exported from the country also yield important NTFPs for local people. A balanced approach is needed between tropical hardwood exploitation and the livelihoods of rural communities, for

which the development and implementation of sustainable forest management plans are essential [9].

Although the value of local ecological knowledge for informing conservation and environmental management is well established [15-17] and there is a general positive association between tree cover and dietary diversity [18], the complex biocultural interactions between tropical forest food resources and the communities they sustain are still understudied [10, 19]. According to Ngome et al. [20], in most poor communities in the Congo Basin, the wide variety of nutrient-rich, wild edible plants are underutilized, either due to ignorance, shame or misconceptions about these foods. Ethnobotanical studies have so far insufficiently provided evidence for the intake, quality and nutritional impacts of WEPs, their contribution to the overall diet and their role in ensuring food security [20]. Considering the increasing conflicts of interests regarding the use of wild resources, accurate assessments of local uses and dependency on wild plants and animals are necessary to evaluate the effects of commercial forest exploitation on both biodiversity and local livelihoods. The aim of this research was to explore the diversity of wild edible plants known and used by the forest-dwelling Baka people in southeast Cameroon. We also evaluated the frequency of their consumption, potential conflicts between local diet and commercial trade in forest resources, and the different field methods to assess of WEP knowledge and consumption.

The Baka are Ubangian-speaking forager-horticulturalists, formerly referred to as 'Pygmies' [21]. Until roughly 50 years ago, they were nomadic foragers who relied on hunting, fishing, gathering, and the exchange of NTFPs for agricultural crops with their neighbors, Bantu-

speaking farmers [22]. Since the 1960s, the Baka have been facing several changes in their livelihood. Due to missionary activities and a government program of sedentarization, they have progressively left their forest camps and settled in villages along logging roads [23]. Nowadays, Baka livelihood is mostly based on the combination of foraging activities, agricultural work in their own fields and wage labor on Bantu farms or for logging companies [22].The settlement programs, which included displacement from protected areas and forced sedentarization, have had drastic effects on Baka livelihood and wellbeing. Increased alcohol abuse, the loss of traditional knowledge, and an increasing destabilization of their natural environment have led to the marginalization of the Baka and their increased dependence on other ethnic groups [24, 25]. To evaluate the Baka's reliance on their surrounding forests in the current situation, it is essential to assess not only their knowledge on wild food plants, but also their actual use and commercialization of these species.

Local knowledge and use of plants are assessed through different ethnobotanical methods, of which the interview is the most widely used. Interview methods vary considerably between studies and are deployed based on the research question addressed [26]. Freelisting is a frequently used interview method, in which informants are asked to list all items they know within a given domain [27, 28]. This technique reveals cultural salience and variations in individuals' topical knowledge [29], and results in a shortlist of highly valued plants [30, 31]. As freelisting enables the collection of data from a large number of informants in a limited amount of time [32], this method is frequently used as a starting point for studying traditional plant knowledge. Local names of plants listed during the freelisting interviews are identified afterwards by collecting voucher specimens for these names. The resulting dataset is then used to

draw conclusions about plant knowledge of a certain group of people and/or the potential contribution of wild plants to their diet [30, 33]. Organizing field trips to collect herbarium specimens of species mentioned during freelisting exercises is often (inaccurately) called the ‘walk-in-the-woods method’ [34, 35]. However, this technique, first coined by Phillips and Gentry in 1993[36], implies that participants are encouraged to actively lead field trips and point out all useful plants they know and/or use [26], instead of only helping the researcher search for specimens that can be linked to the local names mentioned during interviews. Data elicited from freelisting appear to be specific to the context in which they were collected (e.g., in the village), creating an unintended but significant bias in this type of ethnobotanical research [27, 32, 37]. Gathering the data during *ex-situ* interviews, for example in people’s home, away from the ecological context in which people collect their plants, may result in lists of only the most salient plant species. Furthermore, the success of freelisting depends on the informants’ correct understanding of the category or cultural domain (e.g., wild food plants) under discussion [28, 29, 38].

In societies that undergo rapid socio-economic changes, people become more integrated into the market economy, change their lifestyle and adopt cultivated or processed substitutes for wild plants in their diet [39]. This creates a gap between people’s ethnobotanical knowledge and their actual use of plants [40, 41]. A discrepancy between the number of useful species known and those actually used indicates that elders who still know how plants were used in the past do not practice this any longer, and infrequently transfer their skills to the next generation [40]. Freelisting exercises often focus on people’s knowledge [40], while recall surveys, developed by social anthropologists for understanding time allocation [42], lead informants to enumerate what

they have done during a specific period of time. For instance, dietary recall surveys have been developed to estimate the proportion of different food items in people's diet [43]. Recently, dietary recalls were introduced in ethnobotanical studies to assess local uses of plants [44, 45], while income recall surveys have been used to assess the contribution of the sale of different forest products to local livelihoods [46]. Although many studies reported a high diversity of wild edible plant species worldwide [47, 48], research relying on dietary recalls has resulted in surprisingly low numbers of wild species actually being consumed [49, 50]. Dietary recalls carried out in the Democratic Republic of the Congo (DRC) also showed that wild plants did not contribute substantially to rural and urban women diets [51]. Likewise, the dietary recalls that we recently held among the Baka reported only 15 wild edible species [52], which is much less than the list provided recently in the northern Congo area [53] and in stark contrast to the extensive wild plant knowledge documented almost three decades earlier by anthropologists among the same ethnic group [2, 3]. Like freelisting, dietary recalls are limited by the subject's memory [54] and may therefore underreport plant use.

Whether the limited amount of WEP listed by the Baka was a result of a general loss of traditional plant knowledge, a change in food preferences or the effect of the assessment methods, was essential to contextualize our findings. In this study, we explored how different ethnobotanical methods captured the diversity of wild edible plant knowledge and use in a Baka community. We aimed to answer the following questions:

1) Which wild edible plants (WEP) are known and used by the Baka?

2) What are the most frequently consumed WEP species among the Baka?

3) Do commercial logging and NTFP trade affect the availability of wild food plants?

4) How do different methods for assessing WEP use vary in their results?

Materials and Methods

Study site

We collected data on wild food plants in two Baka settlements: Le Bosquet (3°07’38’’N13°52’57’’E) and Kungu (3°02'40"N 14°06’57’’E), Haut Nyong division, southeastern Cameroon. The two settlements, which belong to the same population of Baka hunter-gatherers, are both located at least eight hours by car from the capital Yaoundé, of which four hours on unpaved logging roads (Fig 1). The accessibility of this area highly depends on the weather, as the road quickly deteriorates during the rainy season. The area is covered by a mixture of evergreen and moist semi-deciduous forest within altitudinal ranges of 300–600 meters [55]. In populated areas, the forest cover is largely removed in favor of settlements, cocoa plantations, logging activities and small-scale agriculture. This creates a mosaic of dense primary forest, selectively logged primary forest, secondary forest and agricultural fields, interspersed with trails. The climate of the region is tropical humid, with a major rainy season between late-August and late-November and a major dry season between late-November and mid-March. The annual precipitation reaches about 1500 mm and the average temperature is 25°C [55].

Fig 1. Map of the study area

Before data collection, Free Prior and Informed Consent was obtained from all participants. This study adheres to the Code of Ethics of the International Society of Ethnobiology [56], and received approval from the ethics commission of Leipzig University (196-16/ek) and the Ethical

Committee from the Ministry of Health of Cameroon (n°2018/06/1049/CE/CNERSH/SP). For our inventory of wild food plant knowledge, use and commercialization, we used a combination of four different datasets, obtained from *ex-situ* interviews (freelisting, dietary recalls and income recalls) and *in-situ* ethnobotanical forest surveys ('walk-in-the-woods' method). We collected data in the two Baka settlements in 2018 and 2019, during three different fieldwork periods, to cover variations in wild fruit availability (Table 1).

Table 1.Datasets, fieldwork periods and the different methods used to assess wild food plant knowledge, consumption and commercialization among the two Baka settlements.

Fieldwork period	Feb.-March 2018	Oct.-Nov. 2018	April-May 2019	
Datasets and Methods (number of participants)	major dry season	major rainy season	minor dry season	Data published earlier by authors
1. Freelisting(n=55) *ex-situ*	x			[52]
2. Dietary recalls (n=83) *ex-situ*	x	x	x	[52]
3. Income recalls (n=73) *ex-situ*	x	x		[52]
4. Walk-in-the-woods survey 14 days (n= 20) *in-situ*			x	[71]*

* Only data regarding bush mangoes (Irvingiaceae).

Data collection: *ex-situ* interviews

All data were collected among Baka individuals of 18 years and older. We included all individuals who were present in the settlement and willing to participate. While we aimed to get a gender- and age-balanced sample, we did not exclude any potential participants. All *ex-situ* interviews were held in the French and Baka language by the first author with the assistance of a local research assistant-interpreter. During the 55 freelisting exercises, we asked 24 men and 31 women in the two Baka villages (29 in Le Bosquet, 26 in Kungu, belonging to 51 different households) to report all WEPs they knew [52]. Because no specific term exists in Baka language that refers to 'wild edible plant', we asked them: "Which food from the forest, excluding game, honey and mushrooms, do you know?" (Supplementary File 1). To gather data on the actual consumption of wild food plants and their importance in Baka diet, we conducted a dietary recall protocol that we adapted from the FAO Guidelines for Assessing Dietary Diversity [52, 57]. Participants were asked to list all items they had consumed within the previous 24 hours, and to mention the origin of each food item (from the wild, from agricultural fields or bought at the market). A total of 143 dietary recall interviews were conducted in the two settlements among 83 individuals: 35 men and 48 women (38 in Le Bosquet and 45 in Kungu), belonging to 66 different households; 42 individuals were interviewed once, 22 twice and 11 three times (Supplementary File 1). A detailed account on the outcomes of the freelisting and dietary recalls with regard to cultivated and shop-bought food, preferences and perceptions of wild vs. cultivated food was published earlier [52].

To collect data on wild food plants that were commercialized (either traded as timber or as NTFP), we conducted a 14-day recall survey on the income received through sale, asking 73 participants to list all the items they had sold during this time period [58]. A total of 114

interviews were conducted with43 women and 30 men (34 individuals in Le Bosquet and 39 in Kungu): 32 individuals were interviewed once and 41 twice (Table 1). Part of the *ex-situ* interviews were repeated in other parts of the year to verify whether the consumption and/or commercialization of WEP changed according to the season. None of the three *ex-situ* interview methods were based on a predefined set of species. We did not use pictures of wild food plants, fresh samples or other visual stimuli, and neither gave prompts after informants had listed a certain number of plants.

Data collection: *in-situ* walk-in-the-woods survey

From the local (Baka) names mentioned during the three *ex-situ* interview methods, we constructed a preliminary database of 'folk taxa' of wild plants consumed by the Baka, with tentative scientific names retrieved from literature on Central African wild food plants [2, 3, 6, 59] and earlier ethnographic and ethnoecological data collected between 2012 and 2014 by the first author during ca.18 months of fieldwork in the Baka settlements [22]. During our last fieldwork period (2019), we carried out a walk-in-the-woods survey to obtain botanical specimens to match the Baka names of wild food taxa in our preliminary database, but also to verify whether the Baka knew, consumed or traded more plant species than they mentioned previously during the *ex-situ* interview sessions. Therefore, we asked the Baka to suggest several people of different ages and gender who were knowledgeable on wild edible plants and would agree to join us on our collection trips. We worked with one to four informants on each collection day. In total, we worked with 20 individuals (10 women and 10 men), aged between 29 and 80 years, of which nine had participated previously in the *ex-situ* interviews (two in the dietary and the income recalls; two only in the free listing; five in all three methods). During 14

collection days into the area surrounding Le Bosquet and Kungu, we searched for plants that matched our preliminary list of folk taxa, but also asked our informants to point out any other edible species they saw (Supplementary File 1). When such a plant was encountered, we collected herbarium material using standard botanical methods [27]. Fieldtrips lasted from 7:00 am to 15:00 pm, but the distance covered differed per day, as it depended on the number of plants collected. Many more species were collected in the first few days close to the village, while on the last days, several hours were spent walking to collect rare edible plants. We paid particular attention to clarify information on Baka folk taxa that consisted of more than one botanical species. For most specimens we collected, we asked our informants for 1) the local name (in Baka, French and/or Nzimé, the Bantu language in this area, if known); 2) plant part(s) used; 3) preparation and application methods; 4) when they had last consumed the plant; 5) whether a part of the plant was sold; 6) in the case of trees, whether the wood was commercially logged. We did not limit our ethnobotanical data to plant uses that were shared among our informants, but were keen to record differences in food plant knowledge, consumption frequency and appreciation of wild edibles among informants.

Duplicates of voucher specimens were deposited at the National Herbarium of Cameroon (YA) and at the herbarium of Naturalis Biodiversity Center (L). A third voucher was kept at the study site to verify local names and uses with Baka villagers during group discussions. Plant identification took place at Naturalis, using Central African herbarium specimens and literature, such as the Flora of West Tropical Africa [60], the Flore du Cameroun [61], and monographs on the tropical African flora [62-64]. This literature was also used to verify the vegetation types in which these wild food plants occurred naturally. For species that were difficult to identify, we

consulted botanical experts at Naturalis and abroad. Scientific names were updated using the online portal of Plants of the World Online [65].

Data analysis

We first analyzed the overall diversity of the WEPs reported and collected through the different methods. To assess the general characteristics of wild species consumed by the Baka, information on life form, part used, habitat and commercial timber was categorized in a spreadsheet, after which the distribution of these traits could be quantified. We used the Smith index [66] to assess the saliency of the plant species reported during the free listing interviews. To analyze the actual consumption of wild food plants reported during the dietary recalls, we calculated the citation frequency of the WEPs mentioned as eaten during the previous 24 hours. To analyze consumption data collected during the walk-in-the-woods survey, we categorized the respondents' answers in the following categories: 1) consumed today/yesterday; 2) 3-7 days ago, 3) 2-4 weeks ago; 4) 1-12 months ago; 5) 1-2 years ago; 6) > 2 years ago; and 7) never [52]. We visualized the ranking of the most recently consumed species according to the walk-in-the-woods methods in a bar chart. We also calculated the most frequently sold WEPs from the overall list of plants reported as commercialized in the income survey.

In order to assess whether the full potential of the methods had been used (dietary recalls, freelisting and walk-in-the-woods), species accumulation curves [67] were produced by calculating the cumulative number of species that were reported after interviewing a certain number of informants (freelisting and dietary recalls) and after a certain amount of collection

days (walk-in-the-woods method). Contrary to usual practice, data were not randomized before producing the curves, as several relevant features of the data would have been lost.

Finally, in order to analyze conflicts between commercial timber harvesting and the availability of wild food plants for the Baka, we traced evidence of wild fruit trees felled by timber companies by counting the number of logged tree trunks along the forest trails and on logging trucks passing through the village during 14 days. We cross-referenced wild fruit trees species observed as felled logs and/or said to be cut for commercial timber with the CITES appendices [68] and the IUCN Red List [69] to assess their current conservation status.

Results

Diversity of wild edible plants

A total of 94 folk taxa of WEP were reported during the three different *ex-situ* interview methods and the *in-situ* walk-in-the-woods trips, corresponding to ca. 91 species. The exact number of species is unclear, as eight vouchers could only be identified to genus level and for several West and Central African *Dioscorea* species (wild yams), the taxonomic species delimitation is not clear [70]. Moreover, the Baka recognize different forms within individual yam species and thus some local names refer to the same botanical taxon. In the case of *D. minutiflora,* the Baka distinguish three distinct types: ‘njàkàkà’, ‘báloko’ and ‘kuku’, all with different leaf and tuber morphology. All local and scientific names of each wild edible species, used parts, preparation methods, and the method(s) through which they were recorded are listed in the Supporting Information. The 91 wild edible plant species belonged to 43 different plant families, of which the best represented were Dioscoreaceae (ca. 9 species of wild yams), Irvingiaceae (8 spp.),

Anacardiaceae (5 spp., including 4 species of *Trichoscypha* fruits) and Zingiberaceae (5 spp. of *Aframomum*). Most wild food plants were trees (46%), followed by climbers (27%) including woody lianas and non-woody vines, herbs (19%), shrubs (7%) and ferns (1%). More than half (52.1%) of the wild edible plant species collected by the Baka naturally occurred in primary forest, 31.9% in secondary forests and the rest (16%) in open vegetation. We encountered very little primary forest that was untouched by loggers: the only patch of forest that did not show signs of commercial timber harvesting was dominated by *Gilberiodendron dewevrei*, located at ca. two hours walking distance from Le Bosquet. The selectively logged primary forest, however, contained the majority of the fruit and seed producing primary trees and lianas sought after by the Baka.

Of the 94 folk taxa of wild food plants, 38 were reported by the Baka during the free listing exercises. Initially, 51 local names were mentioned during these interviews, but 13 of those were later excluded because they were either synonyms of Baka plant names that had already been mentioned (three names) or they referred to wild mushrooms (two names), types of honey (six names) or cultivated plants (two names). The most salient WEPs (the most frequently listed first) emerging from the freelisting (Smith index> 0.1) were *Dioscorea burkilliana*, *D. praehensilis*, *D.* cf. *praehensilis* ('ba'), *Irvingia gabonensis*, *Baillonella toxisperma*, and *Panda oleosa*. Of the 83 participants of the dietary recalls, 69 reported having eaten wild plants, and mentioned a total of 12 different species. Two species that emerged from the dietary recalls (*Amaranthus dubius* nd *Raphia* sp.) were not mentioned during the freelisting.

During the dietary recalls, 12 species of wild edible plants emerged, of which the most frequently eaten was *Gnetum* cf. *africanum* (mentioned in 50% of the interviews). The identification to species level of *Gnetum* was difficult due to the absence of flowering material

from male and female individuals during the time of our botanical survey. After *Gnetum* leaves, the second most often consumed were bush mango kernels (Irvingiaceae spp.; 22% of the interviews), *Raphia* palm wine (15%) and several species of wild yams (*Dioscorea* spp.; 15%). Detailed data on the contribution of WEPs to Baka diet compared to cultivated crops and store-bought food were published earlier [52].

During our walk-in-the-woods survey, much more WEP species were reported by the 20 informants as recently eaten. Of the 82 WEP species for which we collected information on last consumption during the walk-in-the-woods survey, 26 were reported as consumed within the last month by at least one informant (Fig2).The most recently consumed was *Solanum erianthum*: the bitter fruits of this shrub were boiled with wild garlic bark (*Afrostyrax lepidophyllus*) and (cultivated) pepper (*Capsicum frutescens*) and taken as a hot drink to wake up in the morning. Moreover, 36 species were eaten within the last 12 months, 11 species between one and two years ago, eight species more than two years ago and one species was never eaten by any of our 20 informants (Fig 2). Of the 26 species consumed within the last month, 23 would not have surfaced from the dietary recalls only, and 16 would have been missed if only the freelisting and dietary recalls would have been carried out. These 16 edible species included two ferns (*Pteridium aquilinum* and *Diplazium sammatii*),three spices (*Xylopia parviflora*, *Olax latifolia* and *Ricinodendron heudelotti*), four fruits (*Passiflora foetida*, *Solanum erianthum*, *Musanga cecropioides* and *Uapaca* cf. *paludosa*), the inner stem of *Laccosperma secundiflorum*, three seeds (*Sterculia oblonga*, *Irvingia robur* and *I. wombulu*), one root (*Renealmia* sp. WTH64), the potable water from the stem of *Tetracera* sp. (WTH42) and the leaves of *Geophila lancistipula*, eaten as a luck charm.

Fig 2.Wild edible species consumed bythe20 informants within the past month.

*Species also reported during freelisting

~Species also reported during dietary recalls

†Species not reported during either freelisting or dietary recalls.

Plant parts used and preparation methods

Different parts of the wild food plants were eaten: most of them were fruits (37% of the species) and seeds (27%), followed by leaves (19%), tubers (12%), bark (5%) and exudate (1%). For 12 species, more than one part was consumed, such as fruits and seeds of *Aframomum* spp., *Irvingia gabonensis*, *Diospyros* cf. *crassiflora*, and *Baillonella toxisperma*; fruits and stem sap of *Musanga cecropioides* and *Chrysophyllum albidum*; fruits and leaves of *Piper umbellatum* and *Olax latifolia*; fruits and roots of *Dioscoreophyllum cumminsii*, and bark and seeds of *Afrostyrax lepidophyllus*. Most WEPs were integrated to cooked meals: only 36% of the 108 edible plant parts were eaten raw, but preparation methods depended on the part(s) consumed (Table 2).

Table 2. Plant parts eaten and preparation methods.

**Part used**	**Fruits**	**Leaves**	**Tubers**	**Seeds**	**Sap**	**Bark**	**Stem**	**Total**
Number of species	38	20	12	31	4	2	1	108*
Eaten raw	31	1	0	3	4	0	0	39
Eaten cooked	7	19	12	25	0	2	1	66
Eaten raw and cooked	0	0	0	3	0	0	0	3

*Total number exceeds the 91 species, as some wild food plants yielded more than one edible part.

The daily meal of the Baka was generally composed by two main dishes: a starchy basis and a stew with a fatty component, leaves, meat or fish. The carbohydrates provided by wild plants came predominantly from wild yams (*Dioscorea* spp.). The collection of these tubers required expert knowledge, as the Baka first verified whether the plant held tubers that were ready to be consumed, which in the case of *D. semperflorens* was done by examining the color of its leaves. The digging of the tubers required certain tools, like a wooden auger to remove the soil and extract the tuber without damaging it, a tool used specifically for both folk taxa of *D. praehensilis*. Once dug out, the tubers were washed and boiled. Some wild yams were only consumed occasionally as an emergency food, like ‘boli’ (*Dioscorea* sp. WTH63), of which the name was translated as ‘adulterer’, referring to the shame felt when eating this tuber. Other occasionally consumed wild tubers provided the Baka with carbohydrates, like those of *Palisota* cf. *barteri* and *Dioscoreophyllum cumminsii*.

To accompany this starchy basis, the Baka prepared a stew in which leafy vegetables and condiments were combined with a fatty basis, mostly obtained from forest seeds. Fruits of *Irvingia* spp., *Baillonella toxisperma* and *Panda oleos* were gathered from the ground and the kernels were taken out. Most seeds were prepared in a similar way: the kernels were dried and/or roasted, and then pounded and/or ground to a powder that was mixed into the stew. Seeds could also be conserved for some time in the form of a pressed, oily cake. Leaves were either cut into thin slices (e.g., *Gnetum* cf. *africanum*) or pounded in a mortar (e.g., *Hilleria latifolia*) and added to the stew. Condiments were grated (*Afrostyrax lepidophyllus* bark), dried (*Aframomum* spp.

seeds), or roasted and crushed (seeds of *Ricinodendron heudelotii* and *Monodora myristica*) before being added to the dish.

Some wild food plants were eaten only on specific occasions, mostly for ritual purposes. To prepare men before hunting expeditions and increase their success, women prepared the leaves of *Anchomanes difformis*, which were thought to make the hunter fearless for elephants, or the rhizome of *Renealmia* sp. (WTH64), which was thought to bring good luck to the hunter. The leaves of *Costus englerianus* or *Adenia mannii* were prepared to strengthening boys after their circumcision.

Commercialization of wild edible plants

During the recall exercise on general income received from sale over the 14 previous days, the Baka reported six different taxa as being sold. The most frequently commercialized NTFP was *Gnetum* cf. *africanum* (26% of the 114 interviews), far more than any of the other wild food plants, which were mentioned as sold in less than 5% of the interviews (seeds and oil of *Irvingia gabonensis, Baillonella toxisperma, Pentaclethra macrophylla,* bark of *Afrostyrax lepidophyllus* and the fruits of *Aframomum* spp., indicated under the general name of 'tondo'). During the walk-in-the-woods surveys, however, the Baka pointed out 24 different WEP species that they sold to middlemen (including the six previously mentioned), mostly in the form of fruits, seeds, or the oil from seeds. Of the 24 commercial species that appeared during the forest surveys, 13 were commonly sold on the international NTFP market (Table 3). When we collected specimens during the walk-in-the-woods surveys, we discovered that the Baka name 'tondo' referred to four different species of *Aframomum*: *A. sceptrum, A. daniellii*, *A. subsericum* and *A.* cf. *longipetiolatum*. Only *A. cereum* was not sold as spice to middlemen, but just consumed within

the household. Of the 24 commercial species traded by the Baka, four appeared in the IUCN Red List as vulnerable and two as near threatened (Table 3).

Table 3. Wild food plant products sold by the Baka and their conservation status. Data were retrieved through different methods.

Species	Plant parts	Walk-in-the-woods	Income recalls	(Inter-) national trade	IUCN status
Afrostyrax lepidophyllus	bark	y	y	[8]	Vulnerable
Irvingia gabonensis	fruits, seeds	y	y	[8]	Near threatened
Panda oleosa	oil from seeds	y		[7]	Least concern
Gnetum cf. *africanum*	leaves	y	y	[8]	Near threatened
Dioscorea cf. *praehensilis*	tuber	y		No data	Least concern
Baillonella toxisperma	fruits, oil from seeds	y	y	Oil [7]	Vulnerable
Pentaclethra macrophylla	oil from seeds	y	y	[7]	Least concern
Garcinia kola	bark	y		[8]	Vulnerable
Piper guineense	fruits	y		[71]	Least concern
Parinari excelsa	firewood	y		No data	Least concern
Xylopia parviflora	fruits	y		[8]	Not evaluated
Irvingia robur	seeds	y		No data	Least concern
Ricinodendron heudelotii	seeds	y		[8]	Vulnerable
Cola acuminata	seeds	y		[8]	Least concern

Tetrapleura tetraptera	fruits	y		[8]	Least concern
Laccosperma secundiflorum	stem (craft material)	y		No data	Least concern
Aframomum cf. *longipetiolatum*	fruits	y	y*	[8]	Not evaluated
Aframomum subsericum	fruits	y	y*	No data	Not evaluated
Aframomum daniellii	fruits	y	y*	No data	Least concern
Aframomum sceptrum	fruits	y	y*	[8]	Not evaluated
Trichoscypha sp. WTH25	fruits	y		[71]	-
Monodora myristica	fruits	y		[71]	Least concern
Piper umbellatum	fruits	y		No data	Not evaluated
Solanum erianthum	fruits	y		No data	Not evaluated

* all mentioned under the Baka folk taxon ‘tondo’.

During our forest walks, we identified six WEP of which the wood was observed as logged or said to be logged by the Baka (Table 4). Three of these species were considered as vulnerable by the IUCN, but none appeared on the CITES Appendices I or II. The moabi tree (*Baillonella toxisperma*), highly valued by the Baka for their fresh fruits and seed oil, was the most sought after by the logging companies operating in Baka territory. Our informants mentioned that only trees exceeding one meter in diameter were felled, so several smaller individuals were still present. However, the extraction of large moabi trees by timber companies strongly affected the amount of fruits and seeds that remained available for the Baka’s subsistence and cash income. Another species that we observed as felled trunks was *Entandrophragma cylindricum*, which itself was inedible, but commonly hosted edible caterpillars, an important food for the Baka. During the forest walks, we also observed several (smaller) trees cut down by the Baka

themselves, mostly to obtain fresh leaves of *Gnetum* cf. *africanum* lianas, to harvest honey, and once to collect the bitter bark of *Garcinia kola*, which was added to *Raphia* palm wine as a flavoring agent.

Table 4. Commercial hardwood tree species producing edible fruits and/or seeds consumed by the Baka, trade names and current conservation status.

Scientific name	Baka name	Commercial trade name	Nr. logs observed*	IUCN status
Baillonella toxisperma	Mabe	Moabi	9	Vulnerable
Chrysophyllum lacourtianum	Bambu	Longhi, Abam		Not evaluated
Diospyros cf. *crassiflora*	Lembe	(Gabon) Ebony	2	Vulnerable
Trichoscypha cf. *abut*	Agbo	-		Least concern
Desbordesia insignis	Ntuo	Alep		Not evaluated
Sterculia oblonga	Egboyo	Eyong		Vulnerable
Afzelia sp.	Tanda	Doussier	3	No data

* Observed during 14 days by the authors. When no logs were observed, the species were mentioned by the Baka as logged by timber companies

Discussion

High diversity in wild food plants

A major insight provided by this study is the high diversity of WEPs known and eaten by the Baka (94 folk taxa, 91 species). Such results are comparable to earlier ethnographic studies carried out among small forest dwelling societies in the Congo Basin [2]. Even though the Baka are facing socio-ecological changes and potential dietary transition [45], our results show that they still know and consume a large diversity of wild edible plants. Apart from the fact that the Baka no longer processed and consumed the oil of *Pentaclethra macrophylla*, but rather sold the seeds to middlemen who processed them elsewhere, we found little evidence for loss of knowledge or disappearing plant uses [52]. Most wild food plants occurred in (selectively logged) primary forests, and consisted predominantly of fruits and seeds of canopy trees and lianas, underlying the importance of this ecosystem for Baka diet and culture. For their daily fat intake, the Baka depended heavily on the large fruits and seeds of the Irvingiaceae family, *Panda oleosa* and *Poga oleosa*. These species are typical to the Congo Basin forests, in which complex food and dispersal interactions take place between big terrestrial mammals (such as elephants) and the local flora [62,72,75]. Many of the oily seed-producing trees in Central Africa are ecological keystone species that are crucial for the survival of local wildlife [72], on which forest-dwelling groups such as the Baka rely on for meat [13].

The diversity of the wild plants eaten by the Baka covers a wide variety of plants parts, highlighting the role played by WEP in providing important nutrients that may not be found in planted crops and store-bought foods. This diversity is also targeted by traders in timber and non-timber forest products: at least 30 WEP species are either sold to middlemen and enter the (inter-) national NTFP market or are extracted by loggers, what sometimes causes conflicts between the Baka and commercial parties entering their territory. The various direct and indirect effects

of logging and trade in NTFPs in this region do not only affect human food resources, but have an impact on the entire ecosystem.

Different methods yield different results

Although our research was performed among a relatively small human population, our results show that different methods resulted in substantial differences in the collected data. The *ex-situ* interviews did not capture the full diversity of WEPs known, used and sold by the Baka. As shown in Fig 3, the species accumulation curve of the freelisting method flattened after interviewing 55 individuals. Typically, 14 of the 55 respondents did not report any WEP, which resulted in several flat sections in the curve.

Fig 3. Species accumulation curves of WEPs mentioned during the 55 freelisting and 83 dietary recall interviews in Le Bosquet and Kungu, southeast Cameroon, 2018.

The species accumulation curve for the dietary recall method flattened after interviewing 83 people (Fig 3). Between respondents 46 and 83, only three new species were mentioned, which suggests that interviewing more respondents would not have led to much more wild edible species being mentioned. Therefore, both freelisting and dietary recalls appeared to have captured most of the WEP diversity that was possible by these methods. In contrast, the species accumulation curve for the walk-in-the-woods method flattened somewhat after 11 days, but not completely (Fig4). Our Baka informants indeed mentioned that there were additional rare species that could only be found after walking for hours in the forest. We know that at least four other edible species could have been found if we had more time to venture further into the forest. From

their Baka names and the literature [2, 59] we assume that these were the African mammee apple (*Mammea africana*) with large edible fruits, a species of *Afzelia* of which the red arils around the seeds were eaten, a species of wild *Raphia* palm of which the sap was fermented into palm wine, and the African walnut tree (*Coula edulis*) that produced highly valued nuts.

Fig 4. Species accumulation curve of wild edible plants mentioned during 14 days of walking in the forest with 20 informants around Le Bosquet and Kungu, southeast Cameroon, 2019.

Regarding the overall WEP diversity recorded through the different methods, we see that the botanical diversity reported would have been considerably less if we would only have conducted the *ex-situ* interviews. The freelisting (38 WEP species) and dietary recalls (12 spp.) resulted in just a fraction of the 91 species found during the walk-in-the-woods survey. Such low number of species reported may be partly due to the fact that not every participant understood the concept of 'wild edible plant'. Wild food plants play an important role in Baka livelihood [2, 3] and knowledge related to edible plants is acquired early during childhood [73]. Therefore, it seems unlikely that those Baka adult informants who did not report any WEP during the freelisting did not know any. They probably did not understand the domain, or the domain was too ubiquitous, a limitation that has been also found in other settings [74].

During the walk-in-the-woods method, the researcher can directly exclude items pointed out by informants that fall outside the domain 'wild edible plant', such as fungi, animal products and cultivated plants, although the latter category can be challenging due to the presence of wild species under various degrees and types of human management and domestication [48]. Local

names listed as ‘wild edible plant’ during *ex-situ* interviews may later, during botanical collection trips, appear to refer to domesticated crops, like the cultivated American crop species tania (*Xanthosoma sagittifolium*) and chili pepper (*Capsicum frutescens*), which were listed as wild edibles during freelisting interviews in the DRC [34]. This was also the case in a recent study on WEP consumed by Baka south of the Dja Reserve [76], in which the domesticated crops okra (*Abelmoschus esculentus*), sorrel (*Hibiscus sabdariffa*) and chili pepper were listed as wild edible plants, as well as an unidentified yam with the Baka name mboto (*Dioscorea* sp.10), which was collected by us and identified as the domesticated *Dioscorea dumetorum*.

Our botanical inventory also revealed that a single folk taxon mentioned during interviews can actually include several species. The local Baka name ‘tondo’ referred to three different species of *Aframomum*, the term ‘bokoko’ to two species of *Klainedoxa*, and ‘payo’ to three different species of *Irvingia* [75]. On the other hand, for the single taxon *Dioscorea minutiflora*, the Baka distinguished in three distinct types. The huge edible plant diversity collected in the forest survey was facilitated by our previously established list of folk taxa. If this preliminary work would not have been conducted, we would have needed more days of fieldwork and more informants to capture the full diversity of WEP consumed by the Baka.

While only six commercial wild food plants were recorded through the income recall surveys, the walk-in-the-woods method revealed 24 traded species, of which several had never been reported previously as sold in Cameroon. Moreover, this *in-situ* method allowed us to observe traces of logged WEP species and unsustainable ways of harvesting NTFPs. Only ten of the 26 species mentioned as recently consumed during the walk-in-the-woods method were reported during the freelisting, while just three of them also emerged through the dietary recalls, although the number of people interviewed during the last two methods was substantially higher. In other

words, 23 recently consumed species would not have been identified with dietary recalls only, and 16 would have been missed if only the freelisting and dietary recalls would have been performed. We speculate that these were plants that were easily forgotten (e.g., spices, condiments, small fruits), species that people feel ashamed of eating (weeds), or items that were previously missed during the freelistings due to misinterpretation of the term ‘wild edible plant’ (e.g., drinking water from lianas, edible latex, ritual food plants). If only using the *ex-situ* interview data, we would have missed four wild fruit trees that were extracted by timber companies. Only two of these WEP-producing commercial hardwoods were mentioned in the freelisting interviews (*Baillonella toxisperma* and *Chrysophyllum lacourtianum*), while only one was recorded through the dietary recalls (*B. toxisperma*).

The advantage of assessing plant knowledge within the ecological context is that many species are encountered that do not pop-up quickly in people’s minds during a (shorter) interview outside the forest. When walking through the natural environment where edible plants occur, it is easier to remember them because of the amounts of visual stimuli to this knowledge at that moment [77]. The walk-in-the-woods method, however, is laborious to perform and requires additional botanical collection, as more rare species will be encountered that are hard to identify, for which the help of taxonomic specialists and support from herbaria is needed. The number of informants that can be taken into the field is also limited and thus forest surveys are not suitable to draw conclusions on the general patterns of foraging, plant knowledge and uses.

On the other hand, *ex-situ* interview methods (including freelisting and dietary recalls) are known to assess the most salient useful plants among a large group of people in a relatively short time, as these techniques are limited by their spatiotemporal context [32, 37]. Freelisting yields much more information than just plant names and saliency, but also shows the items within a

specific (local) domain and consensus in a community [74]. Conducted among a large number of people and in different seasons, dietary and income recall surveys provide a general overview of plants that contribute substantially to people's daily meals and subsistence strategies. Our results indicate, however, that these short *ex-situ* interviews missed out a substantial part of the WEP diversity known, consumed and traded by the Baka. Our insights show the importance of not relying on either *ex-situ* or *in-situ* methods, but rather combining both approaches to maximize the outputs assessing ethnobotanical practices, which was earlier highlighted by Quinlan [74]. Since they can lead to an underestimation of wild edible plants known, consumed and commercially exploited, the implications of studies based solely on *ex-situ* interviews can be grave. Results of such studies may not be representative for the situation on the ground, as trade in NTFPs or conflicts between wild fruit collection and logging of fruit-producing trees may remain invisible. An assessment of Baka knowledge on wild edible plants relying on *ex-situ* methods only might have led to the conclusion that the Baka lost much of their knowledge, or do not use the forest so intensively as their ancestors did. Moreover, the calculations of the contribution of wild plants to local diet and nutrition would have been inaccurate. Several studies based on dietary recalls have concluded that WEP do not play an important role in local diets. Some scholars said they were "confident to provide a fair representation of the dietary contribution of WEP on a population level in our sample" [51:8], even though their botanical collection was limited to finding specimens to match the local names mentioned during their dietary recalls and freelisting interviews. In Brazil, scholars stated after their freelisting and dietary recall surveys that "the low consumption of wild species [….] is notable, which suggests that, in practice, these foods contribute little to contemporary dietary enrichment" [50:337]. Such data could be misused by policy makers, who may conclude that rural communities do not need

the forest that much as previously thought. Considering the importance of wild plants for food security and for providing nutrients that are not present in other foods [78], and the fact that children are major consumers of wild fruits but hardly recruited as interviewees [79, 80], it is crucial to draw the most accurate overview of the diversity of wild food items used by local people, especially in species-rich ecosystems in which plants, animals and human livelihoods are under increasing pressure.

Conclusions

Our results show that choosing an appropriate method (or combination of methods) is essential for assessing wild food plant knowledge and consumption. Ethnobotanical inventories that leave room for informants to point out edible plants that do not appear on predefined lists are essential to capture the full diversity of edible forest species. Our *in-situ* inventories yielded a wide variety of WEP that were not covered by our *ex-situ* interview methods, revealed details on local patterns of foraging and challenged the general ideas of knowledge loss and underutilization of wild food plants in the region. Our mixed methods approach shows the importance of cross-referencing data, not only between different types of interviews, but also between interviews and direct observation during forest trips, for a better assessment of the diversity, consumption frequency, trade and conflicting uses of WEP. We therefore recommend that wild plant knowledge and use should be assessed through the use of a mixed methods approach. Using ‘open’ walk-in-the-woods surveys, in which informants are encouraged to mention any useful plant they know or randomly encounter, after which they are asked when they last used it, provides a wider variety of data. Employing the walk-in-the-woods technique merely to supply specimens for previously composed lists of useful plants from literature or interviews limits the

capacity of this powerful technique to assess wild plant knowledge and use. Freelisting and dietary recalls can be used to provide quantitative data and a more general overview, but researchers should not limit themselves to such methods if they want to capture the full diversity of plants known and used, especially in the case when biased conclusions may have large implications for people's future livelihood, culture and wellbeing.

Acknowledgements

We would like to thank our assistants Appolinaire Ambassa, Ernest Isidore Simpoh and driver Alain Hyppolite Fezeu for their help with the fieldwork. We are grateful to Professor Bonaventure Sonké for his support in Cameroon. David Harris (Edinburgh herbarium); Jan Wieringa, Paul Maasand Carel Jongkind (Naturalis) and Marc Sosef (Meise Botanical Gardens) helped us to identify our specimens. Finally, our greatest gratitude goes to all Baka children, women and men with whom we have lived and worked. Thank you for your trust, hospitality and generous hearts.

References

1. Riddell M. Assessing the impacts of conservation and commercial forestry on livelihoods in northern Republic of Congo. Conserv Soc. 2013; 11:199. doi: 10.4103/0972-4923.121002.
2. Bahuchet S. Dans la forêt d'Afrique Centrale: les Pygmées Baka et Aka. Paris: Peeters Selaf; 1992.
3. Dounias E. Perception and use of wild yams by the Baka hunter-gatherers in south Cameroon. In: Hladik CM, Hladik A, Linares OF, Pagezy H, Semple A, Hadley M, editors.

Tropical forests, people and food: Biocultural interactions and applications to development. Paris: UNESCO-Parthenon; 1993. pp. 621–632.

4. Van Dijk JFW. Non-timber forest products in the Bipindi-Akom region, Cameroon: a socio-economic and ecological assessment. Wageningen: Tropenbos-Cameroon Series 1; 1999.
5. Terashima H, Ichikawa M. A comparative ethnobotany of the Mbuti and Efe Hunter-Gatherers in the Ituri Forest, Democratic Republic of Congo. Afr Study Monogr. 2003; 24:1–168.
6. Yasuoka H. Fledging agriculturalists? Rethinking the adoption of cultivation by the Baka hunter-gatherers. Afr Study Monogr Supplementary Issues 2012; 43: 85–114.
7. Hoare AL. The use of non-timber forest products in the Congo basin: Constraints and opportunities. New York: The Rainforest Foundation; 2007.
8. Ingram V, Schure J. Review of Non Timber Forest Products (NTFPs) in Central Africa. Yaoundé: CIFOR; 2010.
9. Ndoye O, Tieguhong JC. Forest resources and rural livelihoods: the conflict between timber and non-timber forest products in the Congo Basin. Scand J For Res. 2004; 19(S4): 36-44 doi: 10.1080/14004080410034047.
10. Baudron F, Tomscha SA, Powell B, Groot JCJ, Gergel SE, Sunderland T. Testing the various pathways linking forest cover to dietary diversity in tropical landscapes. Front Sustain Food Syst. 2019; 3: 97. doi: 10.3389/fsufs.2019.00097.
11. Shapiro A, Grantham HS, Aguilar-Amuchastegui N, Murray NJ, Gond V, Bonfils D, Rickenbach O. Forest condition in the Congo Basin for the assessment of ecosystem conservation status. BioRxiv [Preprint]. Available from

https://www.biorxiv.org/content/10.1101/2020.03.25.008110v1doi: 10.1101/2020.03.25.008110.

12. De Wasseige C, De Marcken P, Bayol N, Hiol Hiol F, Mayaux P, Desclée B, et al. Les forêts du Bassin du Congo: État des Forêts 2010. Luxemburg: Office des publications de l'Union Européenne; 2012.
13. Fa JE, Currie D, Meeuwig J. Bushmeat and food security in the Congo Basin: Linkages between wildlife and people's future. Environ Conserv. 2003; 30: 71-78.
14. Rist L, Shanley P, Sunderland T, Sheil D, Ndoye O, Liswanti N, et al. The impacts of selective logging on non-timber forest products of livelihood importance. For Ecol Manag. 2012; 268: 57–69. doi: 10.1016/j.foreco.2011.04.037.
15. Chazdon RL, Harvey CA, Komar O, Griffith DM, Ferguson BG, Martínez-Ramos M, et al. Beyond reserves: A research agenda for conserving biodiversity in human-modified tropical landscapes. Biotropica 2009; 41: 142–153. doi: 10.1111/j.1744-7429.2008.00471.x.
16. Pardo-de-Santayana M, Macia MJ. The benefits of traditional knowledge. Nature. 2015; 518: 487–488. doi: 10.4337/9781783474257.iii.19.
17. Pandey AK, Tripathi Y. Ethnobotany and its relevance in contemporary research. J Med Plants Stud. 2017; 5: 123–129.
18. Ickowitz A, Powell B, Salim MA, Sunderland TC. Dietary quality and tree cover in Africa. Glob Environ Change. 2014; 24: 287-294. doi: 10.1016/j.gloenvcha.2013.12.001.
19. Hladik CM, Hladik A, Linares OF, Pagezy H, Semple A, Hadley H. Tropical forests, people and food: Biocultural interactions and applications to development. Paris: UNESCO-Parthenon; 1993.

20. Ngome PIT, Shackleton C, Degrande A, Tieguhong JC. Addressing constraints in promoting wild edible plants' utilization in household nutrition: case of the Congo Basin forest area. Agric Food Sec. 2017; 6(1): 20. doi: 10.1186/s40066-017-0097-5.
21. Robillard M, Bahuchet S. Les Pygmées et les autres: terminologie, catégorisation et politique. J Africanist. 2012; 82(1/2): 15-51.
22. Gallois S, Duda R, Hewlett B, Reyes-García V. Children's daily activities and knowledge acquisition: a case study among the Baka from southeastern Cameroon. J Ethnobiol Ethnomed. 2015; 11: 86. doi: 10.1186/s13002-015-0072-9.
23. Leclerc C. L'adoption de l'agriculture chez les Pygmées Baka du Cameroun: Dynamique sociale et continuité structurale. Versailles: Editions Quae; 2012.
24. Esam Awu H. Conservation-induced resettlement: The case of the Baka of southeast Cameroon - a variation on the habitual mobility–immobility nexus. Transfers. 2016; 6(2): 54–73. doi: 10.3167/TRANS.2016.060205.
25. Ramirez Rozzi FV. Reproduction in the Baka pygmies and drop in their fertility with the arrival of alcohol. PNAS. 2018; 115(27): E6126-E6134.
26. Thomas E, Vandebroek I, Van Damme P. What works in the field? A comparison of different interviewing methods in ethnobotany with special reference to the use of photographs. Econ Bot. 2007; 61: 376–384 doi: 10.1663/0013-0001.
27. Martin GJ. Ethnobotany: A methods manual. London: Earthscan; 2010.
28. Quiroz D, Sosef MSM, Van Andel TR. Why ritual plant use has ethnopharmacological relevance. J Ethnopharmacol. 2016; 188: 48–56. doi: 10.1016/j.jep.2016.05.006.
29. Quinlan M. Considerations for collecting freelists in the field: Examples from Ethobotany. Field methods. 2005; 17: 219–234.doi: 10.1177/1525822X05277460.

30. Mengistu F, Hager H. Wild edible fruit species cultural domain, informant species competence and preference in three districts of Amhara region, Ethiopia. Ethnobot Res App. 2009; 6: 487-502.

31. Ghorbani A, Langenberger G, Sauerborn J. A comparison of the wild food plant use knowledge of ethnic minorities in Naban River Watershed National Nature Reserve, Yunnan, SW China. J Ethnobiol Ethnom. 2012; 8: 17. doi: 10.1186/1746-4269-8-17.

32. Pires de Sousa DC, Taboada Soldati G, Monteiro JM, de Sousa Araújo TA, Albuquerque UP. Information retrieval during free listing is biased by memory: Evidence from medicinal plants. PLoS ONE. 2016; 11: 1–15. doi: 10.1371/journal.pone.0165838.

33. Fongnzossie EF, Nyangono CFB., Biwole AB, Ebai PNB, Ndifongwa NB, Motove J, et al. Wild edible plants and mushrooms of the Bamenda Highlands in Cameroon: Ethnobotanical assessment and potentials for enhancing food security. J Ethnobiol Ethnom. 2020; 16: 1–10. doi: 10.1186/s13002-020-00362-8.

34. Termote C, Van Damme P, Djailo BD. Eating from the wild: Turumbu, Mbole and Bali traditional knowledge on non-cultivated edible plants, District Tshopo, DRCongo. Genet Resour Crop Ev. 2011; 58: 585–618 doi: 10.1007/s10722-010-9602-4.

35. Lulekal E, Asfaw Z, Kelbessa E, Van Damme P. Ethnomedicinal study of plants used for human ailments in Ankober District, North Shewa Zone, Amhara region, Ethiopia. J Ethnobiol Ethnomed. 2013; 9: 63. doi: 10.1186/1746-4269-9-63.

36. Phillips O, Gentry AH. The useful plants of Tambopata, Peru: 1. Statistical hypotheses tests with a new quantitative technique. Econ Bot. 1993; 47: 15–32. doi: 10.1007/BF02862203.

37. Paniagua Zambrana NY, Bussmann RW, Hart RRE, Moya Huanca AL., Ortiz Soria G, Ortiz Vaca MMO., et al. To list or not to list? The value and detriment of freelisting in ethnobotanical studies. Nat Plants. 2018; 4(4): 201–204. doi: 10.1038/s41477-018-0128-7.
38. Cruz Da Cunha LVF, De Albuquerque UP. Quantitative ethnobotany in an Atlantic forest fragment of Northeastern Brazil: Implications to conservation. Environ Monit Assess. 2006; 114: 1–25. doi: 10.1007/s10661-006-1074-9.
39. Kuhnlein HV. Introduction: Why are Indigenous Peoples' food systems important and why do they need documentation? In: Kuhnlein HV, Erasmus B, Spigelski D, editors. Indigenous Peoples' food systems: the many dimensions of culture, diversity and environment for nutrition and health. Rome: FAO; 2009. pp. 1–8.
40. Reyes-García V, Vadez V, Huanca T, Leonard W, Wilkie D. Knowledge and consumption of wild plants: A comparative study in two Tsimane' villages in the Bolivian Amazon. Ethnobot Res Appl. 2005; 3: 201 doi: 10.17348/era.3.0.201-208.
41. De Albuquerque UP. Re-examining hypotheses concerning the use and knowledge of medicinal plants: A study in the Caatinga vegetation of NE Brazil. J Ethnobiol Ethnomed. 2006; 2: 30. doi: 10.1186/1746-4269-2-30.
42. Gross DR. Time allocation: a tool for the study of cultural behavior. Annu Rev Anthropol. 1984; 13(1): 519–558.
43. Munger RG, Folsom AR, Kushi LH, Kaye SA, Sellers TA. Dietary assessment of older Iowa women with a food frequency questionnaire: nutrient intake, reproducibility, and comparison with 24-hour dietary recall interviews. Am J Epidemiol. 199; 2(136): 192-200. doi: 10.1093/oxfordjournals.aje.a116485.

44. Friant S, Ayambem WA, Alobi AO, Ifebueme NM, Otukpa OM, Ogar DA, Alawa CBI, et al. Life on the rainforest edge: food security in the agricultural-forest frontier of Cross River state, Nigeria. Front Sustain Food Syst. 2019; 3: 1–14. doi: 10.3389/fsufs.2019.00113.
45. Reyes-García V, Powell B, Díaz-Reviriego I, Fernández-Llamazares Á, Gallois S, Gueze M. Dietary transitions among three contemporary hunter-gatherers across the tropics. Food Secur. 2019; 11: 1–14. doi: 10.1007/s12571-018-0882-4.
46. Levang P, Lescuyer G, Noumbissi D, Déhu C, Brousolle L, Trees F, Lescuyer G. Does gathering really pay? Case studies from forest areas of the East and South regions of Cameroon. For Trees Livelihoods. 2015; 24(2): 128-143. doi: 10.1080/14728028.2014.1000980.
47. Delang CO. Not just minor forest products: the economic rationale for the consumption of wild food plants by subsistence farmers. Ecol Econ. 2006; 59: 64-73. doi: 10.1016/j.ecolecon.2005.10.006.
48. Bharucha Z, Pretty J. The roles and values of wild foods in agricultural systems. Philos Trans R Soc B: Biological Sciences. 2010; 365: 2913–2926. doi: 10.1098/rstb.2010.0123.
49. Ogle BM. Wild vegetables and micronutrient nutrition: Studies on the significance of wild vegetables in women’s diets in Vietnam. Ph. D. thesis, University of Uppsala. 2001
50. Do Nascimento VT, de Lucena RFP, Maciel MIS, de Albuquerque UP. Knowledge and use of wild food plants in areas of dry seasonal forests in Brazil. Ecol Food Nutr. 2013; 52: 317–343. doi: 10.1080/03670244.2012.707434.
51. Termote C, Bwama Meyi M, Dhed'a Djailo B, Huybregts L, Lachat C, Kolsteren P, Van Damme P. A biodiverse rich environment does not contribute to a better diet: a case study from DR Congo. PLoS ONE. 2012; 7(1): e30533. doi: 10.1371/journal.pone.0030533.

52. Gallois S, Heger WT, van Andel TR, Sonké B, Henry AG. From bush mangoes to bouillon cubes: Wild plants and diet among the Baka, forager-horticulturalists from southeast Cameroon. Econ Bot. 2020; 74: 46-58. doi: 10.1007/s12231-020-09489-x.
53. Betti JL, Kourogue RL, Mbong FA, Fils PEB, Njukouyou OFN. Diversity in the usages of edible wild plants by the Baka and Bakwélé in the periphery of the Tala Forest Management Unit, North Congo. Ethnobot Res Appl. 2020; 19: 1-20.
54. Grandjean AC. Dietary intake data collection: Challenges and limitations. Nutr Rev. 2012; &70: 101–104. doi: 10.1111/j.1753-4887.2012.00545.x.
55. Letouzey R. Notice de la carte phytogéographique du Cameroun au 1:500000: 4. Domaine de la forêt dense humide toujours verte. Toulouse: Institut de la Carte Internationale de la Végétation; 1985.
56. ISE. International Society of Ethnobiology Code of Ethics (with 2008 additions). [2006]. Available from http://ethnobiology.net/code-of-ethics/.
57. Kennedy G, Ballard T, Dop M. Guidelines for measuring household and individual dietary diversity. Rome: FAO; 2011.
58. Godoy R, Reyes-García V, Huanca T, Tanner S, Seyfried C. On the measure of income and the economic unimportance of social capital: Evidence from a native Amazonian society of farmers and foragers. J Anthropol Res. 2007; 63(2): 239-260.
59. Brisson, R. Petit Dictionnaire Baka-Français, Sud Cameroun. Paris: l'Harmattan; 2010.
60. Royal Botanical Gardens Kew. Flora of West Tropical Africa: all territories in West Africa South of Latitude 18° N. and to the West of Lake Chad. Kew: Royal Botanical Gardens; 1931-1973.

61. Association de Botanique Tropicale. Flore du Cameroun. Yaoundé: Herbier National du Cameroun; 1963-2018.

62. Harris DJ. A revision of the Irvingiaceae in Africa. Bull Jard Bot Natl Belg. 1996; 65(1/2): 143-196.

63. Hawthorne WD, Jongkind CCH. Woody plants of western African forests: A guide to the forest trees, shrubs and lianas from Senegal to Ghana. Kew: Royal Botanic Gardens; 2006.

64. Harris DJ, Wortley AH. Monograph of *Aframomum* (Zingiberaceae). Syst Bot Monogr. 2018; 140: 1-204.

65. Plants of the World Online. Royal Botanic Gardens, Kew. [2020]. Available from http://www.plantsoftheworldonline.org.

66. Pennec F, Wencélius J. FLAME 1.1 Free-List Analysis under Microsoft Excel. User's Guide. Paris: CNRS; 2012.

67. Peroni N, Farias H, Araujo P, Hanazaki N. Ecological methods in ethnobotanical and ethnobiological research: Using diversity measurements and richness estimators. In: Albuquerque UP, Cruz da Cunha LVF, Paiva de Lucena RF, Alves RRN, editors. Methods and techniques in ethnobiology and ethnoecology. New York: Springer; 2014. pp. 398–412.

68. CITES. Convention on International Trade in Endangered Species of Wild Fauna and Flora. Appendices I, II, III. [2019]. Available from https://www.cites.org/eng/app/appendices.php

69. IUCN. IUCN Red List of Threatened Species. Version 2020-1. [2020]. Available from https://www.iucnredlist.org.

70. Magwé-Tindo J, Wieringa JJ, Sonké B, Zapfack, L, Vigouroux Y, Couvreur TLP, Scarcelli N. Guinea yam (*Dioscorea* spp., Dioscoreaceae) wild relatives identified using whole plastome phylogenetic analyses. Taxon. 2018; 67(5): 905-915. doi: 10.12705/675.4.

71. Eyog Matig O, Ndoye O, Awono A, Kengue J. Les fruitiers forestiers comestibles du Cameroun. Cotonou: IPGRI; 2006.

72. Beaune D, Fruth B, Bollache L, Hohmann G, Bretagnolle F. Doom of the elephant-dependent trees in a Congo tropical forest. For Ecol Manag. 2013; 295: 109–117. doi: 10.1016/j.foreco.2012.12.041.

73. Gallois S, Duda R, Reyes-García V. Local ecological knowledge among Baka children: a case of “children’s culture”? J Ethnobiol. 2017; 37(1): 60–80. doi: 10.2993/0278-0771-37.1.60.

74. Quinlan M. The free-listing method. In: Liamputtong P, editor. Handbook of research methods in health social sciences. Singapore: Springer International; 2017. pp. 1–16.

75. Gallois, S, Heger, WT, van Andel, TR, Sonké, B, Henry, AG. Comparing apples and pears: The hidden diversity of the Central African bush mangos (Irvingiaceae). Econ Bot. 2020; 74(2): 178-194. doi: 10.1007/s12231-020-09497-x.

76. Billong Fils PE, Afiong Nana N, Betti JL et al. Ethnobotanical survey of wild edible plants used by Baka people in southeastern Cameroon. J Ethnobiol Ethnomed 2020; 16:64 doi: 10.1186/s13002-020-00413-0.

77. Miranda TM, de Mello Amarozo MC, Govone JS, Miranda DM. The influence of visual stimuli in ethnobotanical data collection using the listing task method. Field Methods. 2007; 19: 76–86. doi: 10.1177/ 1525822X06295987.

78. Ong HG, Kim YD. The role of wild edible plants in household food security among transitioning hunter-gatherers: evidence from the Philippines. Food Secur. 2017; 9: 11–24. doi: 10.1007/s12571-016-0630-6.

79. Setalaphruk C, Price L. Children's traditional ecological knowledge of wild food resources: a case study in a rural village in Northeast Thailand. J Ethnobiol Ethnomed. 2007; 3(1): 33. doi: 10.1186/1746-4269-3-33.

80. Crittenden AN, Conklin-Brittain NL, Marlowe FW, Schoeninger MJ, Wrangham R. Foraging strategies and diet composition of Hadza children. Am J Phys Anthropol. 2009; 138(S48): 75–281. doi:10.1002/ajpa.21030.

Supporting information

Supplementary Table 1. Wild edible plants reported during the walk-in-the-woods, free listing and dietary recalls in Le Bosquet and Kungu, southeast Cameroon.

Supplementary File 1. Data collection protocol for *ex-situ* interviews and *in-situ* interviews.

Figure 1

Click here to access/download;Figure;Fig1.tif

Map.tif

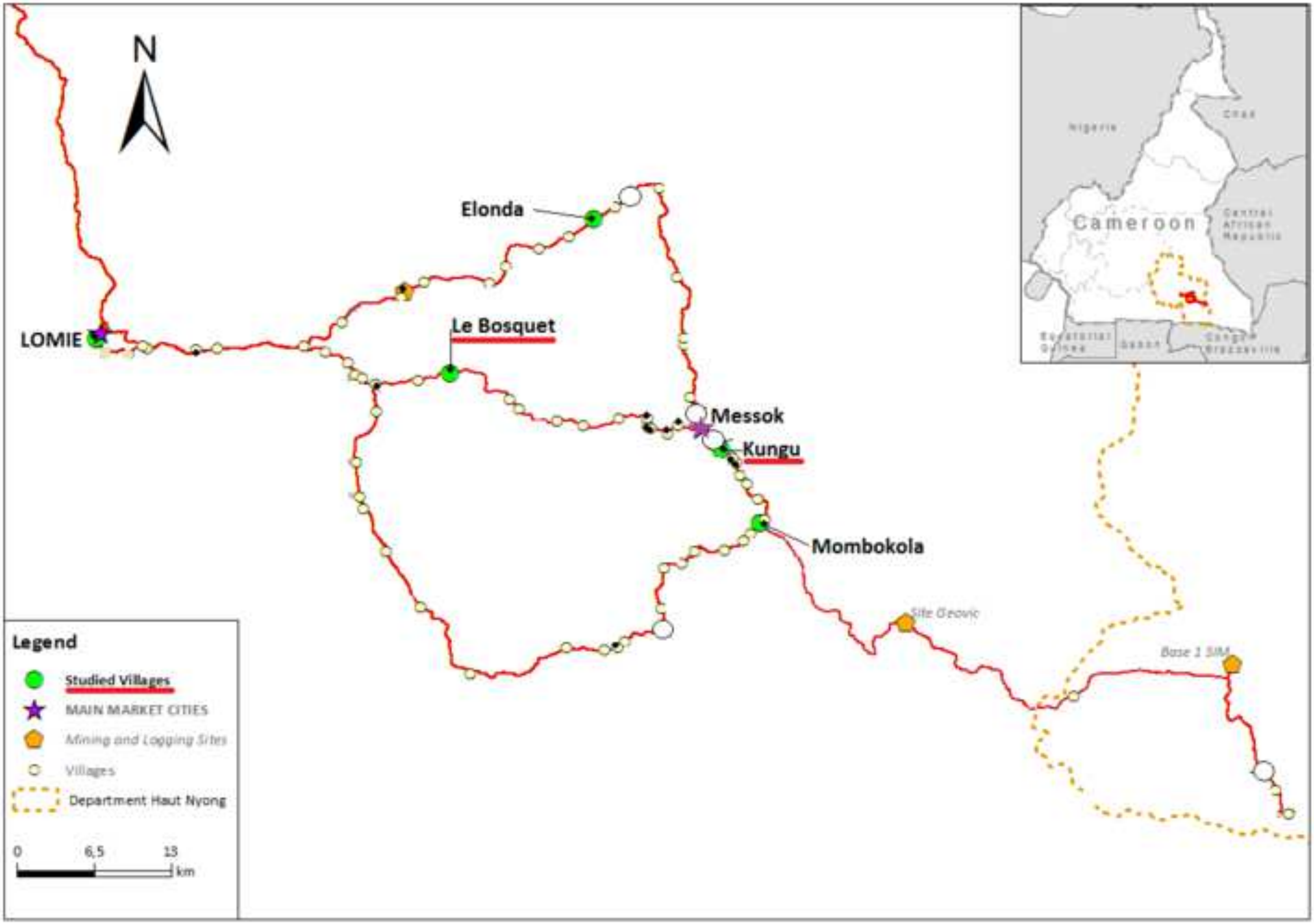

Figure 2

Click here to access/download;Figure;Fig2.tif

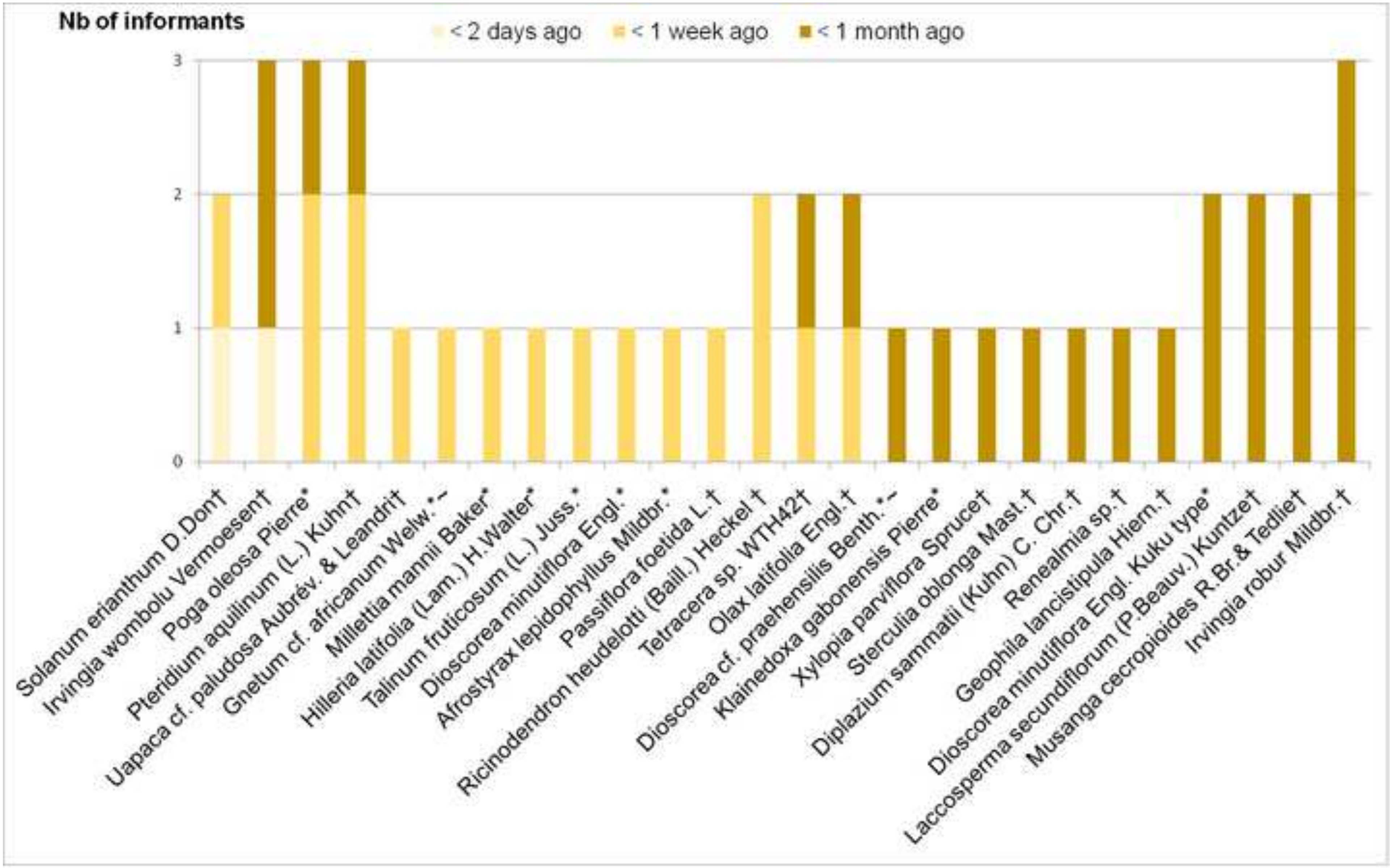

Arri¨ere-plan

Figure 3

Click here to access/download;Figure;Fig3.tif

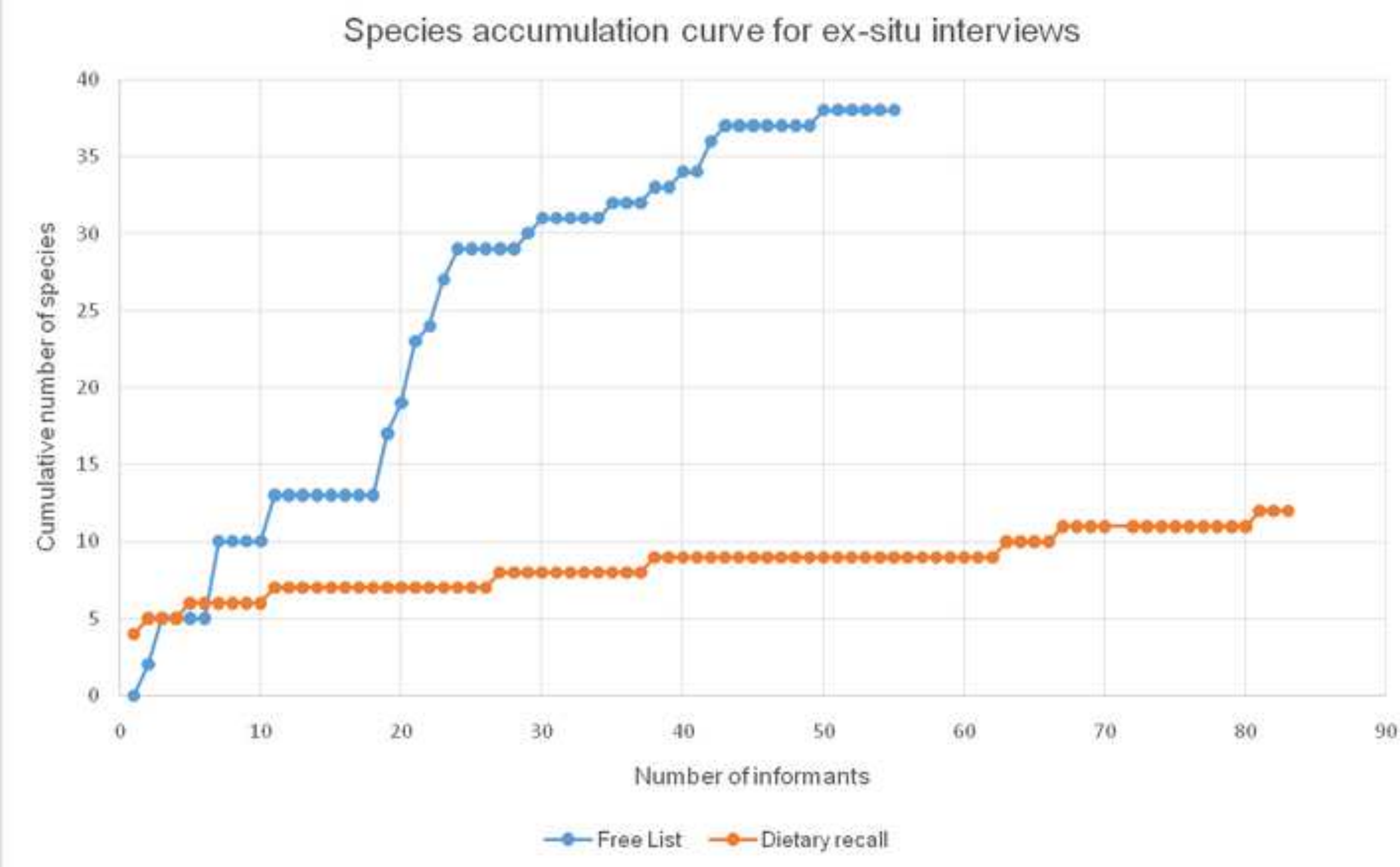

Arriˆ¤re-plan

Figure 4

Click here to access/download;Figure;fig4.tif

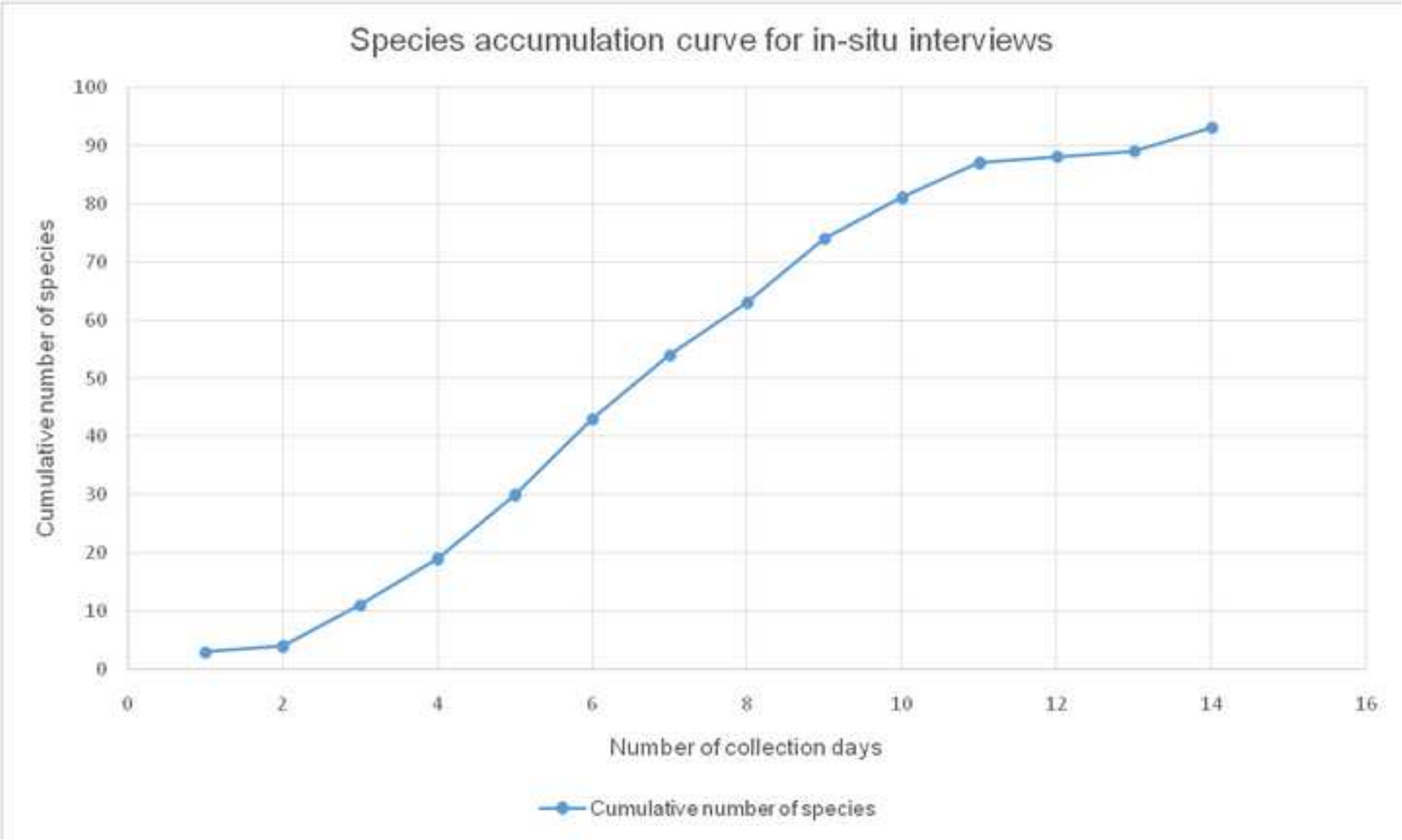

Arri¨re-plan

Supplementary File 1

Click here to access/download
Supporting Information
Supplementary File 1.docx

Supplementary Table 1

Click here to access/download
Supporting Information
Supplementary Table 1.xlsx

~~Importance~~ The importance of choosing appropriate methods for assessing wild food plant knowledge and use: a case study among the Baka in Cameroon.

Short title: Choosing ~~the~~ appropriate methods for assessing wild food plant knowledge.

Sandrine Gallois[1*], Thomas Heger[2], Amanda G. Henry[1], and Tinde van Andel[2,3]

[1]Faculty of Archaeology, Leiden University, Leiden, the Netherlands

[2]Biosystematics Group, Wageningen University, Wageningen, the Netherlands

[3]Naturalis Biodiversity Center, Leiden, the Netherlands

*Corresponding Author:

Sandrine Gallois

Faculty of Archaeology, Leiden University, Leiden, the Netherlands

Email ~~address~~: galloissandrine.ethnoeco@gmail.com

Abstract

In tropical rainforests, access to and availability of natural resources are vital for the dietary diversity and food security of forest-dwelling societies. In the Congo Basin, these are challenged by the increasing exploitation of ~~the~~ forests for bushmeat, commercial hardwood, mining, and large-scale agriculture. In this context, a balanced approach is needed between ~~among~~ the pressures from forest exploitation, non-timber forest product ~~(NTFP)~~ trade and the livelihood and dietary behavior of rural communities ~~is needed~~. While there is a general positive association between ~~forest~~ treecover and dietary diversity, the complex biocultural interactions between tropical forest food resources and the communities they sustain ~~is~~ arestill understudied. This research focuses on the knowledge and use of wild food plants ~~among~~ bythe forest-dwelling Baka people in southeast Cameroon. By using two different sets of methods, namely *ex-situ* interviewsand *in-situ* surveys, ~~we~~ we collected ethnographic and ethnobotanical data ~~among~~ intwo Baka ~~communities~~settlements and ~~in southeastern Cameroon. We aimed to~~ explored the diversity of wild edible plants ~~(WEP)~~ known, the frequency of their consumption, and ~~the~~ potential conflicts between local diet and commercial trade in forest resources. Within a single Baka population,~~We~~ we showed that ~~within a single population, when data on consumption frequency are collected during~~ the *in-situ*walk-in-the-woods method~~,itresults~~ resultedin more detailed information ~~of~~ on~~WEP~~ wild food plantknowledge and use frequency than ~~do~~ the*ex-situ*methods of freelisting~~or~~ anddietary recalls. Our *in-situ* method yielded 91 wild ediblespecies ~~of WEP~~, much more than the *ex-situ*~~methods of~~ freelistinginterviews (38 spp.) and dietary recalls (12 spp.).Our results ~~imply~~suggest that ~~previous~~ studies ~~in the Congo Basin~~ that are based only on *ex-situ*~~surveys~~ interviewsmay ~~have~~ underestimate~~d~~ the importance of ~~WEP~~ wild food plantsfor local communities. We discuss ~~on~~ the limitations and strengths of these different

Formatted: Font: Not Italic

Formatted: Font: Italic

Formatted: Font: Italic

methods for capturing the diversity of ~~WEP~~wild food plantknowledge and uses and ~~. We~~ propose that future studies on ~~WEP~~ wild food plant knowledge and use frequency should rely on a mixed approach~~,~~ that~~tr~~~~combining~~combines*in-situ* and *ex-situ* methods.

Introduction

In tropical rainforests, access to and availability of natural resources are vital for the dietary diversity and food security of forest-dwelling societies. The Congo Basin includes the largest tract of tropical rainforest in Africa. This highly biodiverse region is inhabited by more than 30 million people, including forest-dwelling communities that strongly depend on their natural environment for their survival [1]. A wide variety of wild edible plant species (WEP) has been reported for the Congo Basin, among Bantu-speaking farmers and especially among hunter-gatherers that infrequently practice agriculture [2~~, 3, 4, 5, ~~6]. Local people ~~mainly~~ harvest these wild food plants mainly for subsistence use within the household, but a substantial number of species is locally traded, and a few find their way to global markets [7,8]. The cash income earned with the trade in non-timber forest products (NTFPs),important ~~in~~ forimproving livelihoods, can empower local communities [9]. Wild edible plants ~~may~~ also provide a safety net in times of agricultural shortage and are ~~therefore~~ thusessential for food security [9]. However, ~~the access to and availability of natural resources for forest-dwelling societies in the Congo Basin~~forest-dwelling societiesin the Congo Basinare challenged bya decrease in access to and availability of wild food resources due to the increasing exploitation of the forests for commercial hardwood, precious minerals, and space for oil palm and rubber plantations [10,11]. As a result of large-scale agricultural encroachment, logging, mining and hunting activities, the

ecosystems of this region are under severe pressure~~s~~[~~12, ~~10, 12].A recent survey estimated that just 70% of the standing forests of the Congo Basin remain fully intact [11]. Pressures on local resources have led to a decrease in local people’s access to the important wild plants and bushmeat,~~that ~~whichcontributes substantially to their diet and medicine [13,14]. In Cameroon, for example, more than 60% of the top 23 timber species exported from the country also yield important NTFPs for local people. A balanced approach is needed between tropical hardwood exploitation and the livelihoods of rural communities, for~~is needed, for ~~which the development and implementation of sustainable forest management plans are essential [9].

Although the value of local ecological knowledge ~~in ~~forinforming conservation and environmental management is well established [15~~,16,~~-17] and there is a general positive association between ~~forest ~~treecover and dietary diversity [18], the complex biocultural interactions between tropical forest food resources and the communities they sustain ~~is ~~arestill understudied [~~19, ~~10, 19]. According to Ngomeet al. [20], in most poor communities in the Congo Basin, the wide variety of nutrient-rich, wild edible plants are underutilized, either due to ignorance, shame or misconceptions about these foods. ~~They pointed out that e~~Ethnobotanical studies have so far insufficiently provided evidence ~~on ~~forthe intake, quality and nutritional impacts of WEPs, their contribution to the overall diet and ~~whether they~~their role in~~ensure~~ ensuringfood security [20]. Considering the increasing~~ly~~~~conflicting ~~conflicts ofinterests ~~in~~ regardingthe use of wild resources, accurate assessments of local uses and dependency on wild plants and animals are necessary ~~in order ~~to evaluate the effects of commercial forest exploitation on both biodiversity and local livelihoods. The aim of this research was to explore the diversity of wild edible plants known and used ~~among ~~bythe forest-dwelling Baka people in

southeast Cameroon. We also evaluated the frequency of their consumption, potential conflicts between local diet and commercial trade in forest resources, and the different field methods to assess of WEP knowledge and consumption.

The Baka are Ubangian-speaking forager-horticulturalists, formerly referred to as ‘Pygmies’ [21]. Until roughly 50 years ago, they~~Baka~~ were nomadic foragers~~,~~ who ~~relying~~ relied on hunting, fishing, gathering, and the exchange of NTFPs for agricultural crops with their neighbors, Bantu-speaking farmers [22]. Since the 1960s, the Baka have been facing several changes in their livelihood. Due to missionary activities and a government program of sedentarization, they have progressively left their forest camps and settled in villages along ~~the~~ logging roads [23]. Nowadays, Baka livelihood is mostly based on the combination of foraging activities, agricultural work in their own fields and wage labor on Bantu farms or for logging companies [22].The~~re~~ settlement programs, which included displacement from protected areas and forced sedentarization, have had drastic effects on Baka livelihood and well~~-~~being. Increased alcohol abuse, the~~a~~ loss of traditional knowledge, and an increasing destabilization of their natural environment have led to the marginalization of the Baka and ~~an~~ their increased dependence on other ethnic groups [24, 25]. To evaluate the Baka’s reliance on their surrounding forests in the~~ir~~ current situation, it is essential to assess not only their knowledge on wild food plants, but also their actual use and commercialization of these species.

Local knowledge and use of plants are assessed through different ethnobotanical methods, of which the interview is the most widely used. Interview methods vary considerably between studies and are deployed based on the research question addressed [26]. Freelisting is a

frequently-used interview method, in which informants are asked to list all items they know within a given domain [27, 28]. This technique reveals cultural salience and variations in individuals' topical knowledge [29], and results in a shortlist of highly valued plants [30, 31]. As freelisting~~allows~~ enablesthe collection of data from a large number of informants in a limited amount of time [32], this method is frequently used as a starting point for studying traditional plant knowledge. Local names of plants listed during the freelistinginterviews are identified afterwards by collecting voucher specimens for these names. The resulting dataset is then used to draw conclusions about plant knowledge of a certain group of people and/or the potential contribution of wild plants to their diet [30, 33].Organizing field trips to collect herbarium specimens of species mentioned during freelisting exercises is often (inaccurately) called the 'walk-in-the-woods method' [34, 35]. However, this technique, first coined by Phillips and Gentry in 1993[36], implies that participants are encouraged to actively lead field trips and point out all useful plants they know and/or use [26], instead of only ~~searching~~ helping the researcher searchfor specimens that ~~appear~~ can be linked to ~~on~~ the ~~list of~~ local names ~~derived from~~mentioned during interviews. Data elicited from freelisting appear to be specific to the context in which they were collected (e.g., in the village), creating an unintended but significant bias in this type of ethnobotanical research[27, 32, 37]. Gathering the data during *ex-situ* interviews, for example in people's home, away from the ecological context in which people collect their plants, may result in lists of only the most salient plant species. Furthermore, the success of freelisting depends on the informants' correct understanding of the category or cultural domain (e.g., wild food plants) under discussion [28, 29, 38].

In societies that undergo rapid socio-economic changes, people become more integrated into the market economy, change their lifestyle and adopt cultivated or processed substitutes for wild plants in their diet [39]. This creates a gap between people's ethnobotanical knowledge and their actual use of plants [40, 41]. A discrepancy between the number of useful species known and those actually used indicates that elders who still know how plants were used in the past do not practice this any longer, and infrequently transfer their skills to the next generation [40].

Freelisting exercises often focus on people's knowledge [40], while recall surveys, developed by social anthropologists for understanding time allocation [42], lead informants to enumerate what they have done during a specific period of time. For instance, dietary recall surveys have been developed to estimate the proportion of different food items in people's diet (e.g., [43]).Recently, dietary recalls were introduced in ethnobotanical approach studiesto assess local uses of plants [44, 45], while income recall surveys have been used for toassessing the contribution of the sale of different forest products in tolocal livelihoods [46].

Although many studies reported a high diversity of wild edible plant species worldwide [47, 48], research relying on dietary recalls has resulted in surprisingly low numbers of wild species actually being consumed [49, 50].Dietary recalls carried out in the Democratic Republic of the Congo (DRC) also showed that wild plants did not contribute substantially to rural and urban women diets [51]. Likewise, the dietary recalls that we recently held among the Baka resulted inreported only 15 wild edible species being reported[52], which is much less than the list provided recently in the northern Congo area [53] and in stark contrast to the extensive wild plant knowledge documented almost three decades earlier by anthropologists [2] and [3]amongthe same ethnic group [2, 3]. Like freelisting, dietary recalls are limited by the subject's memory [54] and may therefore underreport plant use.

Whether the limited amount of WEP listed by the Baka was a result of a general loss of traditional plant knowledge, a change in food preferences or the effect of the assessment methods, was essential to contextualize our findings. In this study, we explored how different ethnobotanical methods captured the diversity of wild edible plant knowledge and use ~~among~~ ina Baka community. We aimed to answer the following questions:

1) Which wild edible plants (WEP) are known and used by the Baka?

2) What are the most frequently consumed WEP species among the Baka?

3) ~~May~~ Docommercial logging and NTFP trade affect the availability of wild food plants?

4) How do different methods for assessing WEP use vary in their results?

Materials ~~&~~andMethods

Study site

We collected data on wild food plants in two Baka settlements: Le Bosquet (3°07’38’’N13°52’57’’E) and Kungu (3°02'40"N 14°06’57’’E), Haut Nyong division, southeastern Cameroon. ~~Both~~ The two settlements, which belong to the same population of Baka hunter-gatherers,areboth located at least eight hours by car from the capital Yaoundé, of which four hours ~~are~~ on unpaved logging roads (Fig 1). The accessibility of this area highly depends on the weather, as the road quickly deteriorates during the rainy season. The area is covered by a mixture of evergreen and moist semi-deciduous forest within altitudinal ranges of 300–600 meters [55]. In populated areas, the forest cover is largely removed in favor of settlements, cocoa plantations, logging activities and small-scale agriculture. This creates a mosaic of dense primary

forest, selectively logged primary forest, secondary forest and agricultural fields, interspersed with trails. The climate of the region is tropical humid, with a major rainy season between late-August and late-November and a major dry season between late-November and mid-March. The annual precipitation reaches about 1500 mm and the average temperature is 25°C [55].

Fig 1. Map of the study area

Formatted: Font: Bold

Before data collection, Free Prior and Informed Consent was obtained from all participants. This study adheres to the Code of Ethics of the International Society of Ethnobiology [56],andreceived approval from the ethics committee of Leipzig University (196-16/ek) and the Ethical Committee from the Ministry of Health of Cameroon (n°2018/06/1049/CE/CNERSH/SP).

For our inventory of wild food plant knowledge, use and commercialization, we used a combination of four different datasets, obtained from *ex-situ* interviews (freelisting, dietary recalls and income recalls) and *in-situ* ethnobotanical forest surveys (‘walk-in-the-woods’ method). We collected data in the two Baka settlements in 2018 and 2019, during three different fieldwork periods, to cover variations in wild fruit availability (Table 1).

Table 1.Datasets, fieldwork periods and the different Methods methods used to assess wild food plant knowledge, consumption and commercialization among the two Baka settlements Le Bosquet and Kungu. * Only data regarding bush mango (Irvingiaceae).

Formatted: Font: Bold

Formatted: Font: Bold

Fieldwork period	Feb.-March	Oct.-Nov.	April-May	

	2018	2018	2019	
Datasets and Methods (number of participants)	major dry season	major rainy season	minor dry season	Data published earlier by authors
1. Freelisting(n=55) *ex-situ*	x			[52]
2. Dietary recalls (n=83) *ex-situ*	x	x	x	[52]
3. Income recalls (n=73) *ex-situ*	x	x		[52]
4. Walk-in-the-woods survey 14 days (n= 20) *in-situ*			x	[71]*

* Only data regarding bush mangoes (Irvingiaceae).

Data collection: *ex-situ* interviews

Formatted: Font: Italic

All data were collected among Baka individuals of 18 years and older. We included all individuals who were present in the settlement and willing to participate. While we aimed to get a gender- and age-balanced sample, we did not exclude any potential participants. All *ex-situ* interviews were held in the French and Baka language by the first author with the assistance of a local research assistant-interpreter. During the 55 freelisting exercises, we asked 24 men and 31womenin the two Baka villages(~~belonging to 51 different households:~~29 in Le Bosquet, 26 in Kungu, belonging to 51 different households) to report all WEPs they knew [52]. Because no specific term exists in Baka language that refers to 'wild edible plant', we asked them: "Which

food from the forest, excluding game, honey and mushrooms, do you know?” (Supplementary File 1).

To gather data on the actual consumption of wild food plants and their importance in Baka diet, we conducted a dietary recall protocol that we adapted from the FAO Guidelines for Assessing Dietary Diversity [52, 57]. Participants were asked to list all items they had consumed within the previous 24 hours, and to mention the origin of each food item (from the wild, from agricultural fields or bought at the market). A total of 143 dietary recall interviews were conducted in the two settlements among 83 individuals: 35 men and 48 women(38 in Le Bosquet and 45 in Kungu), belonging to 66 different households~~):~~ ; 42 individuals were interviewed once, 22 twice and 11 three times (Supplementary File 1). A detailed account on the outcomes of the freelisting and dietary recalls with regard to cultivated and shop-bought food, preferences and perceptions of wild vs. cultivated food was published ~~in~~ earlier[52].

To collect data on wild food plants that were commercialized (either traded as timber or as NTFP), we conducted a 14-day recall survey on the income received through sale, asking ~~our~~ 73participants to list all the items they had sold during this time period [58].A total of 114 interviews were conducted with43 women and 30 men (34 individuals in Le Bosquet and 39 in Kungu):32 individuals were interviewed once and 41 twice (Table 1).~~.~~ Part of the *ex-situ* interviews were repeated in other parts of the year to verify whether the consumption and/or commercialization of WEP changed according to the season. None of the three *ex-situ* interview methods were based on a predefined set of species. We did not use pictures of wild food plants, fresh samples or other visual stimuli, and neither gave prompts after informants had listed a certain ~~amount~~ numberof plants.

Data collection: *in-situ* walk-in-the-woods survey

From the local (Baka) names mentioned during the three *ex-situ* interview methods, we constructed a preliminary database of ‘folk taxa’ of wild plants consumed by the Baka, with tentative scientific names retrieved from literature on Central African wild food plants~~ (e.g.,~~[2, 3, 6, 59]~~)~~ and earlier ethnographic and ethnoecological ~~research~~ data collected between 2012 and 2014 ~~among the Baka~~ by the first author ~~[22]~~duringca.18 months of fieldwork ~~spent~~ in the Baka settlements~~, where she collected ethnographic and ethnoecological data~~[22]. During our last fieldwork period(2019), we carried out a walk-in-the-woods survey to obtain botanical specimens to match the Baka names of wild food taxa in our preliminary database, but also to verify whether the Baka knew, consumed or traded more plant species than they mentioned previously during the *ex-situ* interview sessions. Therefore, we asked the Baka to suggest several people of different ages and gender who were knowledgeable on wild edible plants and would agree to join us on our collection trips. We worked with one to four informants on each collection day. In total, we worked with 20 individuals (~~they were~~10 women and 10 men), aged between 29 and 80 years, of which nine had participated previously in the *ex-situ* interviews (two in the dietary and the income recalls; two only in the free listing; five in all three methods). During 14 collection days into the area surrounding Le Bosquet and Kungu, we searched for ~~the species~~plants that matched ~~on~~ our preliminary list of folk taxa, but also asked our informants to point out any other edible ~~plant~~ speciesthey saw (Supplementary File 1). When such a ~~wild edible~~ plant was encountered, we collected herbarium material using standard botanical methods [27]. Fieldtrips lasted from 7:00 am to 15:00 pm, but the distance covered differed per day, as it depended on the number of plants collected. Many more species were collected in the first few days close to the village, while on the last days, several hours were spent walking to collect rare

Formatted: Font: Italic

edible plants. We paid particular attention to clarify information on Baka folk taxa that consisted of more than one botanical species. For most specimens we collected, we asked our informants for 1) the local name (in Baka, French and/or Nzimé, the Bantu language in this area, if known); 2) plant part(s) used; 3) preparation and application methods; 4) when they had last consumed the plant; 5) whether a part of the plant was sold; 6) in the case of trees, whether the wood was commercially logged. We did not limit our ethnobotanical data to plant uses that were shared among our informants, but were keen to record differences in food plant knowledge, consumption frequency and appreciation of wild edibles among informants.

Duplicates of voucher specimens were deposited at the National Herbarium of Cameroon (YA) and at the herbarium of Naturalis Biodiversity Center (L). A third voucher was kept at the study site to verify local names and uses with Baka villagers during group discussions. Plant identification took place at Naturalis, using Central African herbarium specimens and literature, such as the Flora of West Tropical Africa [60], the Flore du Cameroun [61], and monographs on the tropical African flora [62~~, 63, -~~64]. This literature was also used to verify the vegetation types in which these wild food plants occurred naturally. For species that were difficult to identify, we consulted botanical experts at Naturalis and abroad. Scientific names were updated using the online portal of Plants of the World Online [65].

Data analysis

We first analyzed the overall diversity of the WEPs reported and collected through the different methods. To assess the general characteristics of wild species consumed by the Baka, information on life form, part used, habitat and commercial timber was categorized in a spreadsheet, after which ~~pie charts were produced to show~~ the distribution of these traits could be

quantified. We ~~calculated~~ usedthe Smith index [66] to assess the saliency of the plant species reported during the free listing interviews. To analyze the actual consumption of wild food plants reported during the dietary recalls, we calculated the citation frequency of the WEPs mentioned as eaten during the previous 24 hours. To analyze consumption data collected during the walk-in-the-woods survey, we categorized the respondents' answers in the following categories: 1) consumed today/yesterday; 2) 3-7 days ago, 3) 2-4 weeks ago; 4) 1-12 months ago; 5) 1-2 years ago; 6) > 2 years ago; and 7) never [52].~~A bar chart was produced to~~ We visualized the ranking of the most recently consumed species according to the walk-in-the-woods methods in a bar chart. We also calculated the most frequently sold WEPs from the overall list of plants reported as commercialized in the income survey.

In order to assess whether the full potential of the methods had been used(dietary recalls, freelisting and walk-in-the-woods), species accumulation curves [67] were produced by calculating the cumulative number of species that were reported after interviewing a certain number of informants (freelisting and dietary recalls) and after a certain amount of collection days (walk-in-the-woods method).Contrary to usual practice, data were not randomized before producing the curves, as several relevant features of the data would have been lost.

Finally, in order to analyze conflicts between commercial timber harvesting and the availability of wild food plants for the Baka, we traced evidence of wild fruit trees felled by timber companies by counting the number of logged tree trunks along the forest trails and on logging trucks passing through the village during 14 days. We cross-referenced wild fruit trees species observed as felled logs and/or said to be cut for commercial timber with the CITES appendices [68] and the IUCN Red List [69] to assess their current conservation status.

Results

Diversity of wild edible plants

A total of 94folk taxa of WEP were reported during the three different *ex-situ* interview methods and the *in-situ* walk-in-the-woods trips, corresponding to ca. 91 species. The exact number of species is unclear, as eight vouchers could only be identified to genus level and for several West and Central African *Dioscorea*species (wild yams), the taxonomic species delimitation is not clear[70]. Moreover, the Baka recognize different forms within individual yam species and thus some local names refer to the same botanical taxon. In the case of *D. minutiflora*, the Baka distinguish three distinct types: 'njàkàkà', 'báloko' and 'kuku', all with different leaf and tuber morphology. All local and scientific names of each wild edible species, used parts, preparation methods, and the method(s) through which they were recorded are listed in the Supporting Information. lementary Material.

The 91 wild edible plant species belonged to 43 different plant families, of which the best represented were Dioscoreaceae (ca. 9 species of wild yams), Irvingiaceae (8 spp.), Anacardiaceae (5 spp., including 4 species of *Trichoscypha* fruits) and Zingiberaceae (5 spp. of *Aframomum*).MostWEP specieswild food plants were trees (46%), followed by climbers (27%) including woody lianas and non-woody vines, herbs (19%), shrubs (7%) and ferns (1%). More than half (52.1%) of the wild edible plant species collected by the Baka naturally occurred in primary forest, 31.9% in secondary forests and the rest (16%) in open vegetation. We encountered very little primary forest that was untouched by loggers: the only patch of forest that did not show signs of commercial timber harvesting was dominated by

Gilberiodendrondewevrei, located at ca. two hours walking distance from Le Bosquet. The selectively logged primary forest, however, contained the majority of the fruit and seed producing primary trees and lianas sought after by the Baka.

Of the 94folk taxa of ~~WEP~~wild food plants, 38 were reported by the Baka during the free listing exercises. Initially, 51 local names were mentioned during these interviews, but 13 of those were later excluded because they were either synonyms of Baka plant names that had already been mentioned (three names) or they referred to wild mushrooms (two names), types of honey (six names) or cultivated plants (two names). The most salient WEPs (the most frequently listed first) emerging from the freelisting(Smith index> 0.1) were *Dioscoreaburkilliana*, *D. praehensilis*, *D.* cf. *praehensilis* (‘ba’), *Irvingia gabonensis*, *Baillonellatoxisperma*, and *Panda oleosa*. Of the 83 participants of the dietary recalls, 69 reported having eaten wild plants, and mentioned a total of 12 different species. Two species that emerged from the dietary recalls (*Amaranthus dubius*and *Raphia* sp.) were not mentioned during the freelisting.

~~When assessing the WEP that emerged d~~During the dietary recalls, ~~we found~~ 12 species of wild edible plants emerged, of which the most frequently eaten was *Gnetum*cf. *africanum*(mentioned in 50% of the interviews). The identification to species level of *Gnetum* was difficult due to the absence of flowering material from male and female individuals during the time of our botanical ~~fieldwork~~survey. After *Gnetum* leaves, the second most often consumed were bush mango kernels (Irvingiaceae spp.; 22% of the interviews), *Raphia* palm wine (15%) and several species of wild yams (*Dioscorea* spp.; 15%). Detailed data on the contribution of WEPs to Baka diet~~,~~ compared to cultivated crops and store-bought food ~~are~~ werepublished~~in~~ earlier[52].

During our walk-in-the-woods survey, much more WEP species were reported by the 20 informants ~~to have been~~as recently eaten ~~by our informants~~. Of the 82 WEP species for which

Formatted: Font: Not Italic

we collected information on last consumption during the walk-in-the-woods survey, 26 were reported as consumed within the last month by at least one ~~of our 20~~ informant~~s~~ (Fig~~.1~~2).The most recently consumed was *Solanum erianthum*~~.~~: ~~The~~ the bitter fruits of this ~~weedy~~ shrub were boiled with wild garlic bark (*Afrostyraxlepidophyllus*) and (cultivated) ~~hot~~ pepper (*Capsicum frutescens*) and taken as a hot drink to wake up in the morning. Moreover, 36 species were eaten within the last 12 months, 11 species between one and two years ago, eight species more than two years ago and one species was never eaten by any of our 20 informants (Fig 2). Of the 26 species consumed within the last month, 23 would not have ~~been identified~~surfaced~~with~~ from thedietary recalls only, and 16 would have been missed if only the freelisting and dietary recalls would have been ~~performed~~carried out. These 16 edible species ~~were~~ includedtwo ~~edible~~ ferns (*Pteridium aquilinum* and *Diplaziumsammatii*),three spices (*Xylopia parviflora*, *Olax latifolia* and *Ricinodendronheudelotti*), four fruits (*Passiflora foetida*, *Solanum erianthum*, *Musangacecropioides*and *Uapaca* cf. *paludosa*), the inner stem of *Laccospermasecundiflorum*, three seeds (*Sterculia oblonga*, *Irvingia robur*and *I. wombulu*), one root(*Renealmia* sp. WTH64), the potable water from the stem of *Tetracera* sp. (WTH42) and the leaves of *Geophilalancistipula*, eaten as a luck charm.

Fig ~~1~~2.Wild edible species ~~that~~ consumed by~~our~~ the20 informants ~~consumed~~ within the past month.

*Species also reported ~~through~~ duringfreelisting~~;~~

~S~~s~~pecies also reported ~~through~~ duringdietary recalls~~,~~

†~~species~~ Species not reported ~~either in~~duringeither freelisting or dietary recalls.

Formatted: Font: Bold

Formatted: Font: Bold

Plant parts used and preparation methods

Different parts of the wild food plants were eaten: most of them were fruits (37% of the species) and seeds (27%), followed by leaves (19%), tubers (12%), bark (5%) and exudate (1%). For 12 species, more than one part was consumed, such as fruits and seeds of *Aframomum*spp., *Irvingia gabonensis*, *Diospyros* cf. *crassiflora*, and *Baillonellatoxisperma*; fruits and stem sap of *Musangacecropioides*and *Chrysophyllum albidum*; fruits and leaves of *Piper umbellatum* and *Olax latifolia*; fruits and roots of *Dioscoreophyllumcumminsii*, and ~~both~~ bark and seeds of *Afrostyraxlepidophyllus*. Most WEPs were integrated to cooked meals~~. O~~: only 36% of the 108 ~~plant~~ edible plant parts were eaten raw~~(Table 2)~~, ~~while~~ but preparationmethods ~~of preparation~~ depended on the part(s)consumed(Table 2).

Table 2. Plant ~~species,~~ parts eaten and preparation methods.~~*Total number exceeds the 94 species, as some species yield more than one edible part.~~

Formatted: Normal

Formatted Table

Part used	Fruits	Leaves	Tubers	Seeds	Sap	Bark	Stem	Total
Number of species	38	20	12	31	4	2	1	108*
Eaten raw	31	1	0	3	4	0	0	39
Eaten cooked	7	19	12	25	0	2	1	66
Eaten raw and cooked	0	0	0	3	0	0	0	3

*Total number exceeds the 9~~4~~1 species, as some wild food ~~species~~plants yielded more than one edible part.

The daily meal of the Baka was generally composed by two main dishes: a starchy basis and a stew with a fatty component, leaves, meat or fish. The carbohydrates provided by wild plants came predominantly from wild yams (*Dioscorea* spp.). The collection of these tubers implied a set ofrequired expert knowledge, as the Baka first verified whether the plant held tubers that were ready to be consumed, which was done, in the case of *D. semperflorens*was done, by examining the color of its leaves. The digging of the tubers required certain tools, like a wooden auger for toremoving removethe soil and extracting the tuber without damaging it, a tool used specifically for both folk taxa of *D. praehensilis*. Once dug out, the tubers were washed and boiled. Some wild yams were only consumed occasionally as an emergency food, like ‘boli’ (*Dioscorea* sp. WTH63), of which the name means was translated as‘adulterer’, referring to the shame felt when eating itthis tuber. Other, occasionally consumed wild tubers also provided the Baka with carbohydrates, like those of *Palisota* cf. *barteri* and *Dioscoreophyllumcumminsii*.

To accompany this starchy basis, the Baka prepared a stew in which leafy vegetables and condiments were combined with a fatty basis, mostly obtained from forest seeds. Fruits of *Irvingia* spp., *Baillonellatoxisperma* and *Panda oleosa*were gathered from the ground, and the kernels were taken out. Most seeds were prepared in a similar way: the kernels were dried and/or roasted, and then pounded and/or ground to a powder that was mixed into the stew. Seeds could also be conserved for some time in the form of a pressed, oily cake. To the stew, lLeaves were added that were either cut into thin slices (for instancee.g.,*Gnetum*cf.*africanum* leaves) or pounded in a mortar (e.g., *Hilleria latifolia*) and added to the stew. Condiments were grated (bark from *Afrostyraxlepidophyllus*bark), dried (seeds of *Aframomum* spp. seeds), or roasted and crushed (seeds of *Ricinodendronheudelotii* and *Monodoramyristica*) before being added to the dish.

Some wild food plants were eaten only on specific occasions, mostly for ritual purposes. To prepare men before hunting expeditions and increase their success, women prepared the leaves of *Anchomanesdifformis*, which were thought to make the hunter fearless for elephants, or the rhizome of *Renealmia* sp. (WTH64), which was thought to bring good luck to the hunter. The leaves of *Costusenglerianus* or *Adeniamannii*were prepared to strengthening boys after their circumcision.

Commercialization of wild edible plants

During the recall exercise on general income received from sale over the 14 previous days, the Baka reported six different taxa as being sold. The most frequently commercialized NTFP was *Gnetum*cf. *africanum* (26% of the 114 interviews), far more than ~~the~~ anyof the other ~~WEP~~wild food plants, which were mentioned as sold in less than 5% of the interviews (seeds and oil of *Irvingia gabonensis, Baillonella~~B.~~toxisperma, Pentaclethra macrophylla,* bark of *Afrostyraxlepidophyllus*and the fruits of *Aframomum*spp., indicated under the general name of ‘tondo’). During the walk-in-the-woods surveys, however, the Baka pointed out 24 different WEP species that they sold to middlemen (including the six previously mentioned)~~ that they sold to middlemen~~, mostly in the form of fruits, seeds, or the oil from seeds. Of the 24 commercial species that appeared during the forest surveys, 13 were commonly sold on the international NTFP market (Table 3). When we collected specimens during the walk-in-the-woods surveys, we discovered that the Baka name ‘tondo’ referred to four different species of *Aframomum*: *A. sceptrum, A. daniellii, A. subsericum* and *A.* cf. *longipetiolatum*. Only *A. cereum* was not sold as spice to middlemen, but just consumed within the household. Of the 24 commercial species

traded by the Baka, four appeared in the IUCN Red List as vulnerable and two as near threatened (Table 3).

Table 3. Wild food plant products sold by the Baka and their conservation status. Data were retrieved through different methods.

**Species**	**Plant parts**	**Walk-in-the--woods**	**Income recalls**	**(Inter-) national ~~or domestic~~ trade**	**IUCN status**
Afrostyraxlepidophyllus~~Mildbr.~~	bark	y	y	[8]	Vulnerable
Irvingia gabonensis~~(Aubry-Lecomte ex O'Rorke) Baill.~~	fruits, seeds	y	y	[8]	Near threatened
Panda oleosa~~Pierre~~	oil from seeds	y		[7]	Least concern
*Gnetum*cf. *africanum*~~Welw.~~	leaves	y	y	[8]	Near threatened
*Dioscorea*cf. *praehensilis*~~Benth.~~	tuber	y		No data	Least concern
Baillonellatoxisperma~~Pierre~~	fruits, oil from seeds	y	y	Oil [7]	Vulnerable
Pentaclethra macrophylla ~~Benth.~~	oil from seeds	y	y	[7]	Least concern
Garcinia kola ~~Heckel~~	bark	y		[8]	Vulnerable
Piper guineense~~Schumach. &Thonn.~~	fruits	y		[71]	Least concern

Parinariexcelsa~~Sabine~~	firewood	y		No data	Least concern
Xylopia parviflora ~~Spruce~~	fruits	y		[8]	Not evaluated
Irvingia robur~~Mildbr.~~	seeds	y		No data	Least concern
Ricinodendronheudelotii~~(Baill.) Heckel~~	seeds	y		[8]	Vulnerable
Cola acuminata ~~Schott. &Endl.~~	seeds	y		[8]	Least concern
Tetrapleuratetraptera~~(Schum. &Thonn.) Taub.~~	fruits	y		[8]	Least concern
Laccospermasecundiflorum~~(P.Beauv.) Kuntze~~	stem (~~crafts~~craft material)	y		No data	Least concern
*Aframomum*cf. *longipetiolatum*~~Koechlin~~	fruits	y	y*	[8]	Not evaluated
Aframomumsubsericum~~(Oliv. & D. Hanb.) K. Schum.~~	fruits	y	y*	No data	Not evaluated
Aframomumdaniellii~~(Hook.f.) K.Schum.~~	fruits	y	y*	No data	Least concern
Aframomumsceptrum~~(Oliv. &D. Hanb.) K. Schum.~~	fruits	y	y*	[8]	Not evaluated
*Trichoscypha*sp. WTH25	fruits	y		[71]	-
Monodoramyristica~~(Graertm.) Dunal~~	fruits	y		[71]	Least

					concern
Piper umbellatum L.	fruits	y		No data	Not evaluated
*Solanum erianthum*D. Don	fruits	y		No data	Not evaluated

Formatted: Justified

Formatted: Justified

* all mentioned under the Baka folk taxon ‘tondo’.

During our forest walks, we identified six WEP of which the wood was observed as logged or said to be logged by the Baka (Table 4). Three of these species were considered as vulnerable by the IUCN, but none appeared on the CITES Appendixes Appendices I or II. The moabi tree (*BaillonellaB. toxisperma*), highly valued by the Baka for their fresh fruits and seed oil, was the most sought after by the logging companies operating in the Baka territory. Our informants mentioned that only trees exceeding one meter in diameter were felled, so several smaller individuals were still present. The However, the extraction of large moabi trees by timber companies strongly affected the amount of fruits and seeds that remained available for the Baka’s subsistence and cash income. Another species that we observed as felled trunks was *Entandrophragmacylindricum*, which itself was inedible, but commonly hosted edible caterpillars, an important food for the Baka. During the forest walks, we also observed several (smaller) trees cut down by the Baka themselves, mostly to obtain fresh leaves of *Gnetum*cf. *africanum*lianas, to harvest honey, and once to collect the bitter bark of *Garcinia kola*, which is was added to *Raphia* palm wine as a flavoring agent.

Formatted: Font: 12 pt

Table 4. Commercial hardwood tree species producing edible fruits and/or seeds consumed by the Baka, trade names and current conservation status.

Scientific name	Baka name	Commercial trade name	Nr. logs ~~O~~observed* ~~in 14 days~~	IUCN status
Baillonellatoxisperma	Mabe	Moabi	9	Vulnerable
Chrysophyllumlacourtianum	Bambu	Longhi, Abam		Not evaluated
Diospyros cf. *crassiflora*	Lembe	(Gabon) Ebony	2	Vulnerable
*Trichoscypha*cf. *abut*	Agbo	-		Least concern
Desbordesia insignis	Ntuo	Alep		Not evaluated
Sterculia oblonga~~.~~	Egboyo	Eyong		Vulnerable
*Afzelia*sp.	Tanda	Doussier	3	No data

* Observed during 14 days by the authors. When no logs were observed, the species were mentioned by the Baka as logged by timber companies

Discussion

High diversity in wild food plants

A major insight provided by this study is the high diversity of WEPs known and eaten by the Baka, (94 folk taxa, 91 species). Such results are comparable to earlier ethnographic studies carried out among small forest dwelling societies in the Congo Basin ~~(e.g.,~~ [2]~~)~~. Eventhough the Baka are ~~when~~ facing socio-ecological changes and potential dietary transition [45], our results

Formatted: Centered
Formatted: Centered
Formatted: Centered
Formatted: Centered
Formatted: Centered
Formatted: Centered
Formatted: Centered
Formatted: Indent: Left: 0"
Formatted: Not Highlight

show that they~~Baka~~ still know and consume a large diversity of wild edible plants. Apart from the fact that the Baka no longer processed and consumed the oil of *Pentaclethra macrophylla*, but rather sold the seeds to middlemen who processed them elsewhere, we found little evidence ~~of~~ forloss of knowledge or disappearing plant uses [52]. Most wild food plants occurred in (selectively logged) primary forests, and ~~were~~ consistedpredominantly of fruits and seeds of ~~mature~~ canopytrees and lianas, underlying the importance of this ecosystem for Baka diet and culture. For their daily fat intake, the Baka depended heavily on the large fruits and seeds of the Irvingiaceae family, *Panda oleosa* and *Pogaoleosa*. These species are typical to the Congo Basin forests, in which complex food and dispersal interactions take place between big terrestrial mammals (such as elephants) and the local flora [62,72,75]. Many of the oily seed-producing trees in Central Africa are ecological keystone species that are crucial for the survival of local wildlife [72], on which forest-dwelling groups such as the Baka rely on for meat [13].

The diversity of the wild plants eaten by the Baka covers a wide variety of plants parts, highlighting the role played by WEP in providing important nutrients that may not be found in planted crops and store-bought foods. This diversity is also targeted by ~~the~~ traders in timber and non-timber forest products~~, as~~: at least 30 WEP species are either sold to middlemen and enter the (inter-) national NTFP market or are ~~targeted~~ extractedby loggers, ~~underlying~~ which sometimescauses~~the~~ conflicts ~~of~~ between~~wild plant usespresent in~~the Baka and commercial parties entering theirterritory. The various direct and indirect effects of logging and trade in NTFPs in this region ~~may impact~~does not only affect human food resources, but has an impact on the entire ecosystem.

Different methods yield different results

Although our research was performed among a relatively small human population, our results show that different methods resulted in substantial differences in the collected data. The *ex-situ* interviews did not capture the full diversity of WEPs known, used and sold by the Baka~~, even if they efficiently captured the requested information~~. As shown in ~~Figure~~ Fig ~~2~~3, the species accumulation curve of the freelisting method flattened after interviewing 55 individuals. Typically, 14 of the 55 respondents did not report any WEP, which resulted in several flat sections in the curve.~~ The species accumulation curve for the dietary recall method flattened after interviewing 83 people (Fig.2).~~

Fig ~~2~~3. Species accumulation curves of WEPs mentioned during the 55 freelisting and 83 dietary recall interviews in Le Bosquet and Kungu, southeast Cameroon, 2018.

The species accumulation curve for the dietary recall method flattened after interviewing 83 people (Fig 3). Between respondents 46 and 83, only three new species were mentioned, which suggests that interviewing more respondents would not have led to ~~many~~ muchmore wild edible ~~plant~~ species being mentioned. Therefore, both freelisting and dietary recalls appeared to have captured most of the WEP diversity that was possible by these methods. In contrast, the species accumulation curve for the walk-in-the-woods method flattened somewhat after 11 days, but not completely (Fig~~.3~~4). Our Baka informants indeed mentioned that there were additional rare species that could only be found after walking for hours in the forest. We know that at least four other edible species could have been found if we had more time to venture further into the forest. From their Baka names and the literature [2, 59]~~,~~ we assume that these ~~WEP~~ were the African mammee apple (*Mammeaafricana*) with large edible fruits, a species of *Afzelia* of which the red

arils around the seeds ~~are~~ were eaten, a species of wild *Raphia* palm ~~tree~~ of which the sap ~~is~~ was fermented into palm wine, and the African walnut tree (*Coula edulis*) that ~~produces~~ produced highly valued nuts.

~~**Fig 2. Species accumulation curves of WEP mentioned during 55 freelisting and 83 dietary recall interviews in Le Bosquet and Kungu, southeast Cameroon, 2018.**~~

Fig ~~3~~4. Species accumulation curve of ~~WEP~~ wild edible plants mentioned during 14 days of walking in the forest with 20 informants around Le Bosquet and Kungu, southeast Cameroon, 2019.

Regarding the overall WEP diversity ~~gathered~~ recorded through the different methods, we see that the ~~WEP~~ botanical diversity reported would have been considerably less if we would only have conducted the *ex-situ* interviews ~~methods~~. The freelisting (38 WEP species) and dietary recalls (12 spp.) resulted in just a fraction of the 91 species found during the walk-in-the-woods survey. Such low number of species reported may be partly due to the fact that not every participant understood the concept of 'wild edible plant'. Wild food plants play an important role in Baka livelihood [2, 3] and knowledge related to edible plants is acquired early during childhood [73]. Therefore, it seems unlikely that ~~the~~ those Baka adult informants who did not report any WEP during the freelisting did not know any. They probably did not understand the domain, or the domain was too ubiquitous, a limitation that has been also found in other settings [74].

During the walk-in-the-woods method, the researcher can directly exclude items pointed out by informants that fall outside the domain 'wild edible plant', such as fungi, animal products and cultivated plants, although the latter category can be challenging due to the presence of wild species under various degrees and types of human management and ~~intervention through to~~ domestication [48]. ~~Species mentioned as l~~Local names ~~during *ex-situ* interviews~~ listed as 'wild edible plant' during *ex-situ* interviews may later, during botanical collection trips, appear to refer to domesticated crops, like the cultivated American crop species tania (*Xanthosoma sagittifolium*) and chili pepper (*Capsicum frutescens*), which were listed as wild edibles ~~by~~ during freelistinginterviewsin the DRC [34].This was also the case in a recent study on WEP consumed by Baka south of the Dja Reserve [76], in which the domesticated crops okra (*Abelmoschus esculentus*), sorrel (*Hibiscus sabdariffa*) and chili pepper were listed as wild edible plants, as well as an unidentified yam with the Baka name Mbooto (*Dioscorea* sp.10), which was collected by us and identified as the domesticated *Dioscoreadumetorum*.

~~Moreover, o~~Our botanical inventory also revealed that ~~some general terms for~~a single folk ~~taxa~~ taxonmentioned during interviews can actually include~~d~~ several species. The local Baka name 'tondo' referred to three different species of *Aframomum*, the term 'bokoko' to two species of *Klainedoxa*, and 'payo' to three different species of *Irvingia*[75]. On the other hand, for the single ~~taxonomic species~~taxon*Dioscoreaminutiflora*, the Baka distinguished in three distinct types. The huge edible plant diversity collected in the forest survey was facilitated by our previously established list of folk taxa. If this preliminary work would not have been conducted, we would have needed more days of fieldwork and more informants to capture the full diversity of WEP consumed by the Baka.

Formatted: Font: Italic

Formatted: Font: Italic

Formatted: Font: Italic

Formatted: Font: Italic

While only six commercial~~WEP species~~wild food plants were ~~found~~ recordedthrough the income recall surveys, the walk-in-the-woods method revealed 24 traded species, of which several had never been reported previously as sold in Cameroon. Moreover, this *in-situ* method allowed us to observe traces of logged WEP species and unsustainable ways of harvesting NTFPs. ~~Moreover,~~ ~~o~~Only ten of the 26 species mentioned as recently consumed during the walk-in-the-woods method were reported during the freelisting, while just three of them also emerged through the dietary recalls, although the number of people interviewed during the last two methods was substantially higher. In other words, 23 recently consumed species would not have been identified with dietary recalls only, and 16 would have been missed if only the freelisting and dietary recalls would have been performed. We speculate that these were plants that were easily forgotten (e.g., spices, condiments, small fruits), species that people feel ashamed of eating (~~e.g.,~~ weeds), or items that were previously missed during the freelistingsdue to misinterpretation of the term ‘wild edible plant’(e.g., drinking water from lianas, edible latex, ritual food plants) ~~during the free listings~~. ~~Moreover, i~~If only using the *ex-situ* interview data, we would have missed four wild fruit trees that were ~~eaten by the Baka and also~~extracted by timber companies ~~logged~~. Only two of these WEP-producing commercial hardwoods were mentioned in the freelisting interviews (*Baillonellatoxisperma* and *Chrysophyllumlacourtianum*), while only one was recorded through the dietary recalls (*B. toxisperma*).

The advantage of assessing plant knowledge within the ecological context is that many species are encountered that do not pop-up quickly in people’s minds during a (shorter) interview outside the forest. When walking through the natural environment where edible plants occur, it is easier to remember them because of the amounts of visual stimuli to this knowledge at that moment [~~76~~77]. The walk-in-the-woods method, however, is laborious to perform and requires additional

botanical collection, as more rare species will be encountered that are hard to identify, for which the help of taxonomic specialists and support from herbaria is needed. The number of informants that can be taken into the field is also limited and thus forest surveys are not suitable to draw conclusions on the general patterns of foraging, plant knowledge and uses.

On the other hand, *ex-situ* interview methods (including freelisting and dietary recalls) are known to assess the most salient useful plants among a large group of people in a relatively short time, as these techniques are limited by their spatio-temporal context [32, 37].Freelisting yields much more information than just plant names and saliency, but also shows the items within a specific (local) domain and consensus in a community [74].Conducted among a large number of people and in different seasons, dietary and income recall surveys p~~allow to get~~rovide a general overview of ~~the WEP~~plants that contribute substantially to people's daily meals and subsistence strategies. Our results indicate, however, that ~~such~~ theseshort*ex-situ* interviews missed out a substantial part of the WEP diversity known, consumed and traded by the Baka. ~~Therefore, as also reported by [74] regarding the use of freelisting, our~~ Our insights ~~provide evidence of~~show the importance of not relying ~~only~~ on either *ex-situ* or *in-situ* methods, but rather combining both approaches ~~in order~~ to maximize the outputs ~~when aiming to~~ assessing ethnobotanical ~~wild edible plants use and knowledge~~practices, which was earlier highlighted by Quinlan [74].

Since they can lead to an underestimation of wild edible plants known, consumed and commercially exploited, the implications of studies based solely on *ex-situ* interviews can be grave. Results of such studies may not be representative for the situation on the ground, as trade in NTFPs or conflicts between wild fruit collection and logging of fruit-producing trees may remain invisible. ~~The~~ Anassessment of Baka knowledge on wild edible plants relying ~~only~~ on *ex-situ* methods only might have led to the conclusion that the Baka lost much of their knowledge,

or do not use the forest so intensively as their ancestors did. Moreover, the ~~assessment~~ calculationsof the contribution of wild plants to local diet and nutrition ~~may be~~would have been inaccurate. Several studies based on dietary recalls have concluded that WEP do not play an important role in local diets. Some scholars said they were "confident to provide a fair representation of the dietary contribution of WEP on a population level in our sample" [51~~]~~:8], even though their botanical collection was limited to finding specimens to match the local names mentioned during their dietary recalls and freelisting interviews. In Brazil, scholars stated after their freelisting and dietary recall surveys that "~~The~~ the low consumption of wild species [….] is notable, which suggests that, in practice, these foods contribute little to contemporary dietary enrichment" [50:337]~~: 337~~. Such data could be misused by policy makers, who may conclude that rural communities do not need the forest that much as previously thought.

Considering the importance of wild plants for food security and for providing nutrients that are not present in other foods [~~77~~78], and the fact that children are major consumers of wild fruits but hardly recruited as interviewees [~~78~~79, ~~79~~80], it is crucial to draw the most accurate overview of the diversity of wild food items used by local people, especially in species-rich ecosystems in which plants, animals and human livelihoods are under increasing pressure.

Conclusions

Our results show that choosing an appropriate method (or combination of methods) is essential for assessing wild food plant knowledge and consumption. Ethnobotanical inventories that leave room for informants to point out edible plants that do not appear on predefined lists are essential to capture the full diversity of edible forest species. Our *in-situ* inventories yielded a wide variety of WEP that were not covered by our *ex-situ* interview methods, revealed details on local

patterns of foraging and challenged the general ideas of knowledge loss and underutilization of wild food plants in the region.

Our mixed methods approach shows the importance of cross-referencing data, not only between different types of interviews, but also between interviews and direct observation during forest trips, for a better assessment of the diversity, consumption frequency, trade and conflicting uses of WEP. We therefore recommend that wild plant knowledge and use should be assessed through the use of a mixed methods approach. Using 'open' walk-in-the-woods surveys, in which informants are encouraged to mention any useful plant they know or randomly encounter, after which they are asked when they last used it, provides a wider variety of data. Employing the walk-in-the-woods technique merely to supply specimens for previously composed lists of useful plants from literature or interviews limits the capacity of this powerful technique to assess wild plant knowledge and use. Freelisting and dietary recalls can be used to provide quantitative data and a more general overview, but researchers should not limit themselves to such methods if they want to capture the full diversity of plants known and used, especially in the case when biased conclusions may have large implications for people's future livelihood, culture and wellbeing.

Acknowledgements

We ~~firstly~~would like to thank our ~~colleagues, especially our field~~ assistants AppolinaireAmbassa, ~~and~~ Ernest Isidore Simpoh and driver Alain HyppoliteFezeu for their help with the fieldwork. We ~~deeply thank~~are grateful to Professor Bonaventure Sonké for his support in Cameroon~~,~~. David Harris (Edinburgh herbarium), Jan Wieringa, Paul MaasandCarelJongkind (Naturalis) and Marc Sosef (Meise Botanical Gardens)~~,~~ helped us to identify our specimens.

Finally, our greatest gratitude goes to all Baka children, women and men with whom we have lived and worked. Thank you for your trust, hospitality and generous hearts.

References

1. ~~1.~~ Riddell M. Assessing the impacts of conservation and commercial forestry on livelihoods in northern Republic of Congo. ~~Conservation~~ Conserv~~and~~ Soc~~iety~~. 2013;11:199. doi: 10.4103/0972-4923.121002.
2. ~~2.~~ Bahuchet S. Dans la forêt d'Afrique Centrale: les Pygmées Baka et Aka. Paris: PeetersSelaf; 1992.
3. ~~3.~~ Dounias E. Perception and use of wild yams by the Baka hunter-gatherers in south Cameroon. In: Hladik CM, Hladik A, Linares OF, Pagezy H, Semple A, ~~and~~ Hadley M, editors. Tropical ~~Forests~~forests, ~~People~~ people and ~~Food~~food: Biocultural interactions and applications to development. Paris: ~~(~~UNESCO-Parthenon~~)~~; 1993. pp. 621–632.
4. ~~4.~~ Van Dijk JFW. Non-timber forest products in the Bipindi-Akom region, Cameroon: ~~a~~ asocio-economic and ecological assessment. Wageningen: Tropenbos-Cameroon Series 1; 1999.
5. ~~5.~~ Terashima H, Ichikawa M. A comparative ethnobotany of the Mbuti and Efe Hunter-Gatherers in the Ituri Forest, Democratic Republic of Congo. ~~African~~ AfrStudy Monogr~~aphs~~. 2003;24:1–168.
6. ~~6.~~ Yasuoka H. Fledging agriculturalists? Rethinking the adoption of cultivation by the Baka hunter-gatherers. Afr Study Monogr~~African Study Monographs~~ Supplementary Issues~~.~~ 2012;43: 85–114.

Formatted: Font: 12 pt

Formatted: Font: (Default) Times New Roman, 12 pt

Formatted: List Paragraph, Indent: Left: 0", Hanging: 0.3", Numbered + Level: 1 + Numbering Style: 1, 2, 3, … + Start at: 1 + Alignment: Left + Aligned at: 0.25" + Indent at: 0.5", Tab stops: 0.1", Left

Formatted: Font: (Default) Times New Roman, 12 pt, English (United States)

Formatted: Font: (Default) Times New Roman, 12 pt

Formatted: Font: (Default) Times New Roman, 12 pt

Formatted: Font: (Default) Times New Roman, 12 pt

Formatted: Font: (Default) Times New Roman, 12 pt

Formatted: Font: (Default) Times New Roman, 12 pt

Formatted: Font: (Default) Times New Roman, 12 pt

7. ~~7.~~ Hoare AL. The use of non-timber forest products in the Congo basin: Constraints and opportunities. New York: The Rainforest Foundation; 2007.

8. ~~8.~~ Ingram V, Schure J. Review of Non Timber Forest Products (NTFPs) in Central Africa. Yaoundé: CIFOR; 2010.~~.~~

9. ~~9.~~ Ndoye O, Tieguhong JC. Forest resources and rural livelihoods: the conflict between timber and non-timber forest products in the Congo Basin. Scand J For Res~~Scandinavian Journal of Forest Research~~. 2004;19(S4): 36-44 doi: 10.1080/14004080410034047.

10. ~~10.~~ Baudron F, Tomscha SA, Powell B, Groot JCJ, Gergel SE, Sunderland T. Testing the various pathways linking forest cover to dietary diversity in tropical landscapes. Front~~iers inSustainable~~ SustainFood Syst~~ems~~. 2019;3: 97. doi: 10.3389/fsufs.2019.00097.

11. ~~11.~~ Shapiro A, Grantham HS, Aguilar-Amuchastegui N, Murray NJ, Gond V, Bonfils D, ~~and~~ Rickenbach O. Forest condition in the Congo Basin for the assessment of ecosystem conservation status. BioRxiv [Preprint]. ~~2020 bioRxiv2020.03.25.008110. [posted 2020 March 26].~~ Available from https://www.biorxiv.org/content/10.1101/2020.03.25.008110v1~~https://www.biorxiv.org/content/10.1101/2020.03.25.008110v1.abstract.~~doi: 10.1101/2020.03.25.008110.

12. ~~12.~~ De Wasseige C, De Marcken P, Bayol N, HiolHiol F, Mayaux P, Desclée B, et al. Les ~~Forêts~~ forêts du Bassin du Congo: État des Forêts 2010. Luxemb~~o~~urg: Office des publications de l'Union Européenne; 2012.

13. ~~13.~~ Fa JE, Currie D, Meeuwig J. Bushmeat and food security in the Congo Basin: ~~linkages~~ Linkages between wildlife and people's future. ~~Environmental~~ Environ Conserv~~ation~~. 2003;30: 71-78.

Formatted: Font: (Default) Times New Roman, 12 pt

Formatted: Font: (Default) Times New Roman, 12 pt

Formatted: Font: (Default) Times New Roman, 12 pt

Formatted: Font: (Default) Times New Roman, 12 pt

Formatted: Font: (Default) Times New Roman, 12 pt

14. 14. Rist L, Shanley P, Sunderland T, Sheil D, Ndoye O, Liswanti N, et al. The impacts of selective logging on non-timber forest products of livelihood importance. Forest ForEcology Ecoland Management. 2012;268: 57–69. doi: 10.1016/j.foreco.2011.04.037.

15. 15. Chazdon RL, Harvey CA, Komar O, Griffith DM, Ferguson BG, Martínez-Ramos M, et al. Beyond reserves: A research agenda for conserving biodiversity in human-modified tropical landscapes. Biotropica. 2009;41: 142–153. doi: 10.1111/j.1744-7429.2008.00471.x.

16. 16. Pardo-de-Santayana M, Macia MJ. The benefits of traditional knowledge. Nature. 2015;518: 487–488. doi: 10.4337/9781783474257.iii.19.

17. 17. Pandey AK, Tripathi Y. Ethnobotany and its relevance in contemporary research. Journal ofJ Medicinal Plants Studies. 2017;5: 123–129.

18. 18. Ickowitz A, Powell B, Salim MA, Sunderland TC. Dietary quality and tree cover in Africa. Global Environmental Change. 2014;24: 287-294. doi: 10.1016/j.gloenvcha.2013.12.001.

19. 19. Hladik CM, Hladik A, Linares OF, Pagezy H, Semple A, Hadley H. Tropical Forestsforests, People people and Foodfood: Biocultural interactions and applications to development. Paris: UNESCO-Parthenon; 1993.

20. 20. Ngome PIT, Shackleton C, Degrande A, Tieguhong JC. Addressing constraints in promoting wild edible plants' utilization in household nutrition: case of the Congo Basin forest area. Agriculture and Food Security. 2017;6(1): 20. doi: 10.1186/s40066-017-0097-5.

21. 21. Robillard M, Bahuchet S. Les Pygmées et les autres: terminologie, catégorisation et politique. Journaldes Africanistes. 2012;82(82 1/2): 15-51.

Formatted: Font: (Default) Times New Roman, 12 pt, Spanish (Spain)

Formatted: Font: (Default) Times New Roman, 12 pt

Formatted: Font: (Default) Times New Roman, 12 pt

Formatted: Font: (Default) Times New Roman, 12 pt

Formatted: Font: (Default) Times New Roman, 12 pt

Formatted: Font: (Default) Times New Roman, 12 pt

22. ~~22.~~ Gallois S, Duda R, Hewlett B, Reyes-García V. Children's daily activities and knowledge acquisition: a case study among the Baka from southeastern Cameroon. ~~Journal of~~ JEthnobiol~~ogyand~~ Ethnomed~~icine~~. 2015;11: 86. doi: 10.1186/s13002-015-0072-9.

23. ~~23.~~ Leclerc C. L'adoption de l'agriculture chez les Pygmées Baka du Cameroun: Dynamique sociale et continuité structurale. Versailles: Editions Quae; 2012.

24. ~~24.~~ EsamAwu H. Conservation-induced resettlement: The case of the Baka of southeast Cameroon - a variation on the habitual mobility–immobility nexus. Transfers. 2016;6(2): 54–73. doi: 10.3167/TRANS.2016.060205.

25. ~~25.~~ Ramirez Rozzi FV. Reproduction in the Baka pygmies and drop in their fertility with the arrival of alcohol. PNAS. 2018;115(27): E6126-E6134.

26. ~~26.~~ Thomas E, Vandebroek I, Van Damme P. What works in the field? A comparison of different interviewing methods in ethnobotany with special reference to the use of photographs. ~~Economic~~ EconBot~~any~~. 2007;61: 376–384 doi: 10.1663/0013-0001.

27. ~~27.~~ Martin GJ. Ethnobotany: A methods manual. London: Earthscan; 2010.

28. ~~28.~~ Quiroz D, Sosef MSM, Van Andel TR. Why ritual plant use has ethnopharmacological relevance. J~~ournalof~~ Ethnopharmacol~~ogy~~. 2016;188: 48–56. doi: 10.1016/j.jep.2016.05.006.

29. ~~29.~~ Quinlan M. Considerations for collecting freelists in the field: Examples from Ethnobotany. Field methods. 2005;17: 219–234 doi: 10.1177/1525822X05277460.

30. ~~30.~~ Mengistu F, Hager H. Wild edible fruit species cultural domain, informant species competence and preference in three districts of Amhara region, Ethiopia. Ethnobot~~any~~ Res~~earch and~~ App~~lications~~. 2009; 6: 487-502.

31. ~~31.~~ Ghorbani A, Langenberger G, Sauerborn J. A comparison of the wild food plant use knowledge of ethnic minorities in Naban River Watershed National Nature Reserve,

Formatted: Font: (Default) Times New Roman, 12 pt, English (United States)

Formatted: Font: (Default) Times New Roman, 12 pt

Formatted: Font: (Default) Times New Roman, 12 pt, English (United States)

Formatted: Font: (Default) Times New Roman, 12 pt

Formatted: Font: (Default) Times New Roman, 12 pt

Formatted: Font: (Default) Times New Roman, 12 pt

Yunnan, SW China. J~~ournal of~~Ethnobiol~~ogyand~~ Ethnom~~edicine~~. 2012;8: 17. doi: 10.1186/1746-4269-8-17.

32. ~~32.~~ Pires de Sousa DC, Taboada Soldati G, Monteiro JM, de Sousa Araújo TA, Albuquerque UP. Information retrieval during free listing is biased by memory: Evidence from medicinal plants. ~~PloS~~ PLoSONE. 2016;11: 1–15. doi: 10.1371/journal.pone.0165838.

33. ~~33.~~ Fongnzossie EF, Nyangono CFB., Biwole AB, Ebai PNB, Ndifongwa NB, Motove J, et al. Wild edible plants and mushrooms of the Bamenda Highlands in Cameroon: ~~ethnobotanical~~ Ethnobotanical assessment and potentials for enhancing food security. J~~ournalof~~ Ethnobiol~~ogyand~~ Ethnomedi~~cine~~. 2020;16: 1–10. doi: 10.1186/s13002-020-00362-8.

34. ~~34.~~ Termote C, Van Damme P, Djailo BD. Eating from the wild: Turumbu, Mbole and Bali traditional knowledge on non-cultivated edible plants, District Tshopo, DRCongo. Genet~~ic~~Resour~~cesand~~ Crop Ev~~olution~~. 2011;58: 585–618 doi: 10.1007/s10722-010-9602-4.

35. ~~35.~~ Lulekal E, Asfaw Z, Kelbessa E, Van Damme P. Ethnomedicinal study of plants used for human ailments in Ankober District, North Shewa Zone, Amhara region, Ethiopia. J~~ournal of~~Ethnobiol~~ogyand~~ Ethnomed~~icine~~. 2013;9: 63. doi: 10.1186/1746-4269-9-63.

36. ~~36.~~ Phillips O, Gentry AH. The useful plants of Tambopata, Peru: 1. Statistical hypotheses tests with a new quantitative technique. Econ~~omic~~ Bot~~any~~. 1993;47: 15–32. doi: 10.1007/BF02862203.

37. ~~37.~~ Paniagua Zambrana NY, Bussmann RW, Hart RRE, Moya Huanca AL., Ortiz Soria G, Ortiz Vaca MMO., et al. To list or not to list? The value and detriment of freelisting in ethnobotanical studies. Nat~~ure~~ Plants. 2018; 4(4): 201–204. doi: 10.1038/s41477-018-0128-7.

Formatted: Font: (Default) Times New Roman, 12 pt

Formatted: Font: (Default) Times New Roman, 12 pt

Formatted: Font: (Default) Times New Roman, 12 pt, Spanish (Spain)

Formatted: Font: (Default) Times New Roman, 12 pt

Formatted: Font: (Default) Times New Roman, 12 pt, English (United States)

Formatted: Font: (Default) Times New Roman, 12 pt

~~1.~~ ~~38.~~ Cruz Da Cunha LVF, De Albuquerque UP. Quantitative ethnobotany in an Atlantic forest fragment of Northeastern Brazil: Implications to conservation. Environ~~mental~~Monit~~oringand~~ Assess~~ment~~. 2006;114: 1–25. doi: 10.1007/s10661-006-1074-9.

38.

39. ~~39.~~Kuhnlein HV. Introduction: Why are Indigenous Peoples' food systems important and why do they need documentation? In: Kuhnlein HV, Erasmus B, Spigelski D, editors. Indigenous Peoples' food systems: the many dimensions of culture, diversity and environment for nutrition and health~~, ed~~. ~~(~~Rome: FAO~~)~~; 2009. pp. 1–8.

40. ~~40.~~ Reyes-García V, Vadez V, Huanca T, Leonard W, Wilkie D. Knowledge and consumption of wild plants: A comparative study in two Tsimane' villages in the Bolivian Amazon. Ethnobot~~any~~ Res~~earchand~~ Appl~~ications~~. 2005;3: 201 doi: 10.17348/era.3.0.201-208.

41. ~~41.~~ De Albuquerque UP. Re-examining hypotheses concerning the use and knowledge of medicinal plants: A study in the Caatinga vegetation of NE Brazil. J~~ournalof~~ Ethnobiol~~ogyand~~ Ethnomed~~icine~~. 2006;2: 30. doi: 10.1186/1746-4269-2-30.

42. ~~42.~~ Gross DR. Time allocation: a tool for the study of cultural behavior. Annu~~alr~~Rev~~iewof~~ ~~a~~Anthropol~~ogy~~. 1984;13(1): 519–558.

43. ~~43.~~ Munger RG, Folsom AR, Kushi LH, Kaye SA, Sellers TA. Dietary assessment of older Iowa women with a food frequency questionnaire: nutrient intake, reproducibility, and comparison with 24-hour dietary recall interviews. Am~~erican~~ J~~ournalof~~ Epidemiol~~ogy~~. 199;2(136): 192-200. doi: 10.1093/oxfordjournals.aje.a116485

Formatted: Font: (Default) Times New Roman, 12 pt, Spanish (Spain)

Formatted: Font: (Default) Times New Roman, 12 pt

Formatted: Font: (Default) Times New Roman, 12 pt

Formatted: List Paragraph, Indent: Left: 0", Hanging: 0.3", Numbered + Level: 1 + Numbering Style: 1, 2, 3, … + Start at: 1 + Alignment: Left + Aligned at: 0.25" + Indent at: 0.5", Tab stops: 0.1", Left

Formatted: Font: (Default) Times New Roman, 12 pt

Formatted: Font: (Default) Times New Roman, 12 pt

Formatted: Font: (Default) Times New Roman, 12 pt, French (France)

Formatted: Font: (Default) Times New Roman, 12 pt

44. ~~44.~~ Friant S, Ayambem WA, Alobi AO, Ifebueme NM, Otukpa OM, Ogar DA, Alawa CBI, et al. Life on the rainforest edge: food security in the agricultural-forest frontier of Cross River state, Nigeria. Front~~iersin~~ Sustain~~able~~ Food Syst~~ems~~. 2019;3: 1–14. doi: 10.3389/fsufs.2019.00113.

45. ~~45.~~ Reyes-García V, Powell B, Díaz-Reviriego I, Fernández-Llamazares Á, Gallois S, Gueze M. Dietary transitions among three contemporary hunter-gatherers across the tropics. Food Secur~~ity~~. 2019;11: 1–14. doi: 10.1007/s12571-018-0882-4.

46. ~~46.~~ Levang P, Lescuyer G, Noumbissi D, Déhu C, Brousolle L, Trees F, Lescuyer G. Does gathering really pay ? Case studies from forest areas of the East and South regions of Cameroon. For Trees Livelihoods. 2015;24(2): 128-143. doi: 10.1080/14728028.2014.1000980.

47. ~~47.~~ Delang CO. Not just minor forest products: the economic rationale for the consumption of wild food plants by subsistence farmers. Ecol~~ogical~~ Econ~~omics~~. 2006;59: 64-73. doi: 10.1016/j.ecolecon.2005.10.006.

48. ~~48.~~ Bharucha Z, Pretty J. The roles and values of wild foods in agricultural systems. Philos~~ophical~~ Trans~~actionsof the Royal~~R Soc~~iety~~ B: Biological Sciences. 2010;365: 2913–2926. doi: 10.1098/rstb.2010.0123.

49. ~~49.~~ Ogle BM. Wild vegetables and micronutrient nutrition: Studies on the significance of wild vegetables in women's diets in Vietnam. Ph. D. thesis, University of Uppsala~~: Acta Universitatis Upsaliensis;~~, 2001

50. ~~50.~~ Do Nascimento VT, de Lucena RFP, Maciel MIS, de Albuquerque UP. Knowledge and use of wild food plants in areas of dry seasonal forests in Brazil. Ecol~~ogyof f~~Food ~~and n~~Nutr~~ition~~. 2013;52: 317–343. doi: 10.1080/03670244.2012.707434.

Formatted: Font: (Default) Times New Roman, 12 pt, Dutch (Netherlands)

Formatted: Font: (Default) Times New Roman, 12 pt

Formatted: Font: (Default) Times New Roman, 12 pt

Formatted: Font: (Default) Times New Roman, 12 pt

Formatted: Font: (Default) Times New Roman, 12 pt

Formatted: Font: (Default) Times New Roman, 12 pt

Formatted: Font: (Default) Times New Roman, 12 pt

51. ~~51.~~ Termote C, BwamaMeyi M, Dhed'aDjailo B, Huybregts L, Lachat C, Kolsteren P, Van Damme P. A biodiverse rich environment does not contribute to a better diet: a case study from DR Congo. PLoS ONE. 2012;7(1): e30533. doi: 10.1371/journal.pone.0030533.

52. ~~52.~~ Gallois S, Heger WT, van Andel TR, Sonké B, Henry AG. From bush mangoes to bouillon cubes: Wild plants and diet among the Baka, forager-horticulturalists from southeast Cameroon. Econ~~omic~~ Bot~~any~~. 2020;74: ~~, 1-13~~46-58. doi: 10.1007/s12231-020-09489-x.

53. ~~53.~~ Betti JL, Kourogue RL, Mbong FA, Fils PEB, Njukouyou OFN. Diversity in the usages of edible wild plants by the Baka and Bakwélé in the periphery of the Tala Tala Forest Management Unit, North Congo~~. ~~.Ethnobot~~any~~ Res~~earchand~~ Appl~~ications~~. 2020;~~-~~19: 1-20.

54. ~~54.~~ Grandjean AC. Dietary intake data collection: Challenges and limitations. Nutr~~ition~~Rev~~iews~~. 2012;70: 101–104. doi: 10.1111/j.1753-4887.2012.00545.x.

55. ~~55.~~ Letouzey R. Notice de la carte phytogéographique du Cameroun au 1:500000: 4. Domaine de la forêt dense humide toujours verte. Toulouse: Institut de la Carte Internationale de la Végétation; 1985.

56. ~~56.~~ ISE. International Society of Ethnobiology Code of Ethics (with 2008 additions). [2006]. Available from http://ethnobiology.net/code-of-ethics/.

57. ~~57.~~ Kennedy G, Ballard T, Dop M. Guidelines for measuring household and individual dietary diversity. Rome: ~~Food and Agriculture Organization of the United Nations~~ ~~(~~FAO~~)~~;2011.

58. ~~58.~~ Godoy R, Reyes-García V, Huanca T, Tanner S, Seyfried C. On the measure of income and the economic unimportance of social capital: Evidence from a native Amazonian society of farmers and foragers. J~~ournalof~~ Anthropol~~ogical~~Res~~earch~~. 2007;63(2): 239-260.

Formatted: Font: (Default) Times New Roman, 12 pt

Formatted: Font: (Default) Times New Roman, 12 pt

59. ~~59.~~ Brisson, R. Petit Dictionnaire Baka-Français, Sud Cameroun. Paris:l'Harmattan; 2010.

60. ~~60.~~ Royal Botanical Gardens Kew. Flora of West Tropical Africa: all territories in West Africa South of Latitude 18° N. and to the West of Lake Chad. Kew: Royal Botanical Gardens; 1931-1973.

61. ~~61.~~ Association de Botanique Tropicale. Flore du Cameroun. Yaoundé: Herbier National du Cameroun; 1963-2018.

62. ~~62.~~ Harris DJ. A revision of the Irvingiaceae in Africa. Bull~~etindu~~ Jard~~inbotanique~~ Bot ~~national~~ Natl ~~de Belgique/Bulletin van de Nationale Plantentuin van Belgie~~Belg. 1996;65(1/2): 143-196.

63. ~~63.~~ Hawthorne WD, Jongkind CCH. Woody plants of western African forests~~. ~~:A guide to the forest trees, shrubs and lianas from Senegal to Ghana. Kew: Royal Botanic Gardens; 2006.

64. ~~64.~~ Harris DJ, Wortley AH. Monograph of *Aframomum* (Zingiberaceae). Syst~~ematic~~ Bot~~any~~Monogr~~aphs~~. 2018;140: 1-204.

65. ~~65.~~ Plants of the World Online. ~~Facilitated by the~~ Royal Botanic Gardens, Kew. [2020]. Available from http://www.plantsoftheworldonline.org.

66. ~~66.~~ Pennec F, Wencélius J. FLAME 1.1 Free-List Analysis under Microsoft Excel. User's Guide. Paris: CNRS; 2012.

67. ~~67.~~ Peroni N, Farias H, Araujo P, Hanazaki N. Ecological ~~Methods~~ methods in ~~Ethnobotanical~~ ethnobotanical and ~~Ethnobiological~~ ethnobiological ~~Research~~research: Using diversity measurements and richness estimators. In: Albuquerque UP, Cruz da Cunha LVF, Paiva de Lucena RF, Alves RRN, editors. Methods and techniques in ethnobiology and ethnoecology. ~~Humana Press.~~New York: Springer; 2014. pp. 398–412.

Formatted: English (United States)

Formatted: Dutch (Netherlands)

Formatted: Font: Italic

Formatted: Font: (Default) Times New Roman, 12 pt

Formatted: Font: (Default) Times New Roman, 12 pt

Formatted: Font: (Default) Times New Roman, 12 pt

Formatted: Font: (Default) Times New Roman, 12 pt

Formatted: Font: (Default) Times New Roman, 12 pt

Formatted: Font: (Default) Times New Roman, 12 pt

Formatted: Font: (Default) Times New Roman, 12 pt

Formatted: Font: (Default) Times New Roman, 12 pt

68. ~~68.~~ CITES. Convention on International Trade in Endangered Species of Wild Fauna and Flora. Appendices I, II, III. [2019]. Available from https://www.cites.org/eng/app/appendices.php

69. ~~69.~~ IUCN. ~~The~~ IUCN Red List of Threatened Species. Version 2020-1. [2020]. Available from https://www.iucnredlist.org.

70. ~~70.~~ Magwé-Tindo J, Wieringa JJ, Sonké B, Zapfack, L, Vigouroux Y, Couvreur TLP, Scarcelli N. Guinea yam (*Dioscorea* spp., Dioscoreaceae) wild relatives identified using whole plastome phylogenetic analyses. Taxon. 2018;67(5): 905-915. doi: 10.12705/675.4.

71. ~~71.~~ EyogMatig O, Ndoye O, Awono A, Kengue J. Les fruitiers forestiers comestibles du Cameroun. Cotonou: IPGRI; 2006.

~~2.~~ ~~72.~~ Beaune D, Fruth B, Bollache L, Hohmann G, Bretagnolle F. Doom of the elephant-dependent trees in a Congo tropical forest. For~~est~~Ecol~~ogyand~~ Manag~~ement~~. 2013;295: 109–117. doi: 10.1016/j.foreco.2012.12.041.

72.

~~3.~~ ~~73 .~~ Gallois S, Duda R, Reyes-García V. Local ecological knowledge among Baka children: a case of “children’s culture”? J~~ournalof~~ Ethnobiol~~ogy~~. 2017;37(1): 60–80. doi: 10.2993/0278-0771-37.1.60.

73.

~~4.~~ ~~74.~~ Quinlan M. The free-listing method. In: Liamputtong P, editor. Handbook of research methods in health social sciences. Singapore: Springer International; 2017. pp. 1–16.

74.

Formatted: Font: (Default) Times New Roman, 12 pt

Formatted: Font: (Default) Times New Roman, 12 pt

Formatted: Font: (Default) Times New Roman, 12 pt, Italic

Formatted: Font: (Default) Times New Roman, 12 pt

Formatted: Font: 12 pt

Formatted: Font: Times, 12 pt

Formatted: Font: (Default) Times New Roman, 12 pt

Formatted: Font: (Default) Times New Roman, 12 pt

Formatted: List Paragraph, Indent: Left: 0", Hanging: 0.3", Numbered + Level: 1 + Numbering Style: 1, 2, 3, … + Start at: 1 + Alignment: Left + Aligned at: 0.25" + Indent at: 0.5", Tab stops: 0.1", Left

Formatted: Font: (Default) Times New Roman, 12 pt

Formatted: List Paragraph, Indent: Left: 0", Hanging: 0.3", Numbered + Level: 1 + Numbering Style: 1, 2, 3, … + Start at: 1 + Alignment: Left + Aligned at: 0.25" + Indent at: 0.5", Tab stops: 0.1", Left

Formatted: Font: (Default) Times New Roman, 12 pt

Formatted: List Paragraph, Indent: Left: 0", Hanging: 0.3", Numbered + Level: 1 + Numbering Style: 1, 2, 3, … + Start at: 1 + Alignment: Left + Aligned at: 0.25" + Indent at: 0.5", Tab stops: 0.1", Left

5. 75. Gallois, S, Heger, WT, van Andel, TR, Sonké, B, Henry, AG. Comparing apples and pears: The hidden diversity of the Central African Bush bush Mangos mangos (Irvingiaceae). EconomicBotany. 2020; 74(2): 178-194. doi: 10.1007/s12231-020-09497-x.

75.

76. 76.BillongFilsPE, Afiong NanaN, Betti JL et al. Ethnobotanical survey of wild edible plants used by Baka people in southeastern Cameroon.J EthnobiolEthnomed2020;16:64 doi: 10.1186/s13002-020-00413-0.

77. Miranda TM, de Mello Amarozo MC, Govone JS, Miranda DM. The influence of visual stimuli in ethnobotanical data collection using the listing task method. Field Methods. 2007;19: 76–86. doi: 10.1177/ 1525822X06295987.Miranda TM, de Mello Amarozo MC, Govone JS, Miranda DM. The Influence of Visual Stimuli in Ethnobotanical Data Collection Using the Listing Task Method. Field methods. 2007; 19: 76–86. doi: 10.1177/ 1525822X06295987.

6.78.

7. 77. Ong HG, Kim YD. The role of wild edible plants in household food security among transitioning hunter-gatherers: evidence from the Philippines. Food Security. 2017;9: 11–24. doi: 10.1007/s12571-016-0630-6.

79.

8. 78. Setalaphruk C, Price L. Children's traditional ecological knowledge of wild food resources: a case study in a rural village in Northeast Thailand. Journal ofEthnobiologyand Ethnomedicine. 2007;3(1): 33. doi: 10.1186/1746-4269-3-33.

80.

Formatted: Font: (Default) Times New Roman, 12 pt

Formatted: Font: (Default) Times New Roman, 12 pt

Formatted: Font: (Default) Times New Roman, 12 pt

Formatted: List Paragraph, Indent: Left: 0", Hanging: 0.3", Numbered + Level: 1 + Numbering Style: 1, 2, 3, … + Start at: 1 + Alignment: Left + Aligned at: 0.25" + Indent at: 0.5", Tab stops: 0.1", Left

Formatted: Font: (Default) Times New Roman, 12 pt, English (United States)

Formatted: English (United States)

Formatted: Font: Not Italic

Formatted: English (United States)

Formatted: Font: Italic

Formatted: English (United States)

Formatted: English (United States)

Formatted: English (United States)

Formatted: No underline, Font color: Auto, English (United States)

Formatted: Font: (Default) Times New Roman, 12 pt, No underline, Font color: Auto

Formatted: Font: (Default) Times New Roman, 12 pt

Formatted: Normal, Indent: Left: -0.3"

Formatted: Font: (Default) Times New Roman, 12 pt, No underline, Font color: Auto

Formatted: Font: (Default) Times New Roman, 12 pt

Formatted: List Paragraph, Indent: Left: 0", Hanging: 0.3", Numbered + Level: 1 + Numbering Style: 1, 2, 3, … + Start at: 1 + Alignment: Left + Aligned at: 0.25" + Indent at: 0.5", Tab stops: 0.1", Left

Formatted: Font: (Default) Times New Roman, 12 pt, No underline, Font color: Auto

Formatted: Font: (Default) Times New Roman, 12 pt

Formatted: List Paragraph, Indent: Left: 0", Hanging: 0.3", Numbered + Level: 1 + Numbering Style: 1, 2, 3, … + Start at: 1 + Alignment: Left + Aligned at: 0.25" + Indent at: 0.5", Tab stops: 0.1", Left

~~9.~~ ~~79.~~Crittenden AN, Conklin-Brittain NL, Marlowe FW, Schoeninger MJ, Wrangham R. Foraging strategies and diet composition of Hadza children. Am~~erican~~ J~~ournalof~~ Phys~~ical~~Anthropol~~ogy~~. 2009;138(S48): 75–281. doi:10.1002/ajpa.21030.

81.

~~80.Betti JL, Yongo OD, Mbomio DO, Iponga DM, Ngoye A. An ethnobotanical and floristical study of medicinal plants among the Baka Pygmies in the periphery of the Ipassa-Biosphere reserve, Gabon. European Journal of Medicinal Plants. 2013; 3(2): 174-205. doi:10.9734/EJMP/2013/2550.~~

~~Supplementary Material~~Supporting information

Supplementary Table 1. Wild edible plants reported during the walk-in-the-woods, free listing and dietary recalls in Le Bosquet and Kungu, southeast Cameroon.

Supplementary File 1. Data collection protocol for *ex-situ* interviews and *in-situ* interviews.~~WTH = acronym of collector Willem Thomas Heger; SG = Sandrine Gallois. Voucher specimens were deposited at the National Herbarium of Cameroon (YA) and Naturalis Biodiversity Center (L). * only "tondo", the general Baka term for *Aframomum* sp. was reported in the income survey. ** identifications based on Baka names and literature [2, 59, 80].~~

Formatted: Font: (Default) Times New Roman, 12 pt, No underline, Font color: Auto

Formatted: Font: (Default) Times New Roman, 12 pt

Formatted: List Paragraph, Indent: Left: 0", Hanging: 0.3", Numbered + Level: 1 + Numbering Style: 1, 2, 3, … + Start at: 1 + Alignment: Left + Aligned at: 0.25" + Indent at: 0.5", Tab stops: 0.1", Left

Formatted: Font: (Default) Times New Roman, 12 pt, No underline, Font color: Auto

Formatted: Font: Italic, No underline, Font color: Auto

Formatted: Font: Italic, No underline, Font color: Auto

Formatted: Font: Bold

Error! Unknown document property name.
Leiden University, Leiden, the Netherlands
[illegible]

Anna Fodor
Academic Editor
PLOS ONE

Leiden, 6 January, 2021

Dear Dr. Fodor,

Thank you for allowing us to adapt our manuscript PONE-D-20-37238, entitled “The importance of choosing appropriate methods for assessing wild food plant knowledge and use: a case study among the Baka in Cameroon”. We have followed your suggestions and hope that the manuscript now fits the submission standards of PLoS ONE.

We have submitted a revised version of our manuscript labeled as “revised manuscript with track changes” and a clean copy (unmarked) labeled as “manuscript”, as well as the response to the Editor's comments, Dr Muhammad Ishtiaq, in the document "Response to Reviewer" with a point by point answers.

Below, we list each point made by the academic editor, and our respective answer.

Additional Editor Comments:

Paper needs followings changes/improvements and data provisions; in order to proceed it for further.
(1) needs to improve English language, there are many mistakes in the article.(letter of English experts reviewed as supporting needed.

> **Answer to comment #1:** Our manuscript was checked by someone with ample experience in publishing scientific articles in the English language.

(2) Letter of Ethical and ISE and other institution permission letters are required.

> **Answer to comment #2:** The International Society for Ethnobiology does not issue permission letters and does not have an ethnical board: it just published standards for researchers, to which we adhered. However, our research was carried out within the ERC project “HARVEST: Plant Foods in Human Evolution; Factors affecting the harvest of nutrients from the floral environment”. For this research project, our co-author Amanda Henry received approval from the Ethical Board of the Faculty of Medicine of the University of Leipzig, Germany. We have added this letter to our submitted files and listed the HARVEST project in the funding. Moreover, before we started our fieldwork, we received permission from the National Committee of Ethics for the Research of Human Health (CNERSH) of the Cameroonian government. We also added this letter to the submitted files.

(3) The references cited in text and reference section needs to be rechecked as it has many mistakes in it.

Answer to comment #3: We have checked all references in the text and reference list and adapted them to the style of PLoS ONE.

(4) Figure of study area needed.

Answer to comment #4: We included a map of the study area (Figure 1)

(5) Model of questionnaire needed.

Answer to comment #5: We included the model of our questionnaire in our Supporting Information (Supplementary file 1).

(6) Families names are not correct, recheck and resend table 1.

Answer to comment #6: Family and author names of the species are now listed in the Supporting Information (Supplementary Table 1). All scientific names were checked and updated suing Plants of the World Online.

(7) Provide ubiquitous herbarium numbers for all plants collected. it has two types of numbering.

Answer to comment #7: Herbarium numbers for all plants collected are listed in Supplementary Table 1, but there are two acronyms (WTH and SG), referring to the two main collectors: Willem Thomas Heger and Sandrine Gallois. We had to follow the Naturalis guidelines of specimen collection for this study.

(8) Figures are dim/blurred, send high density/good quality figures.

Answer to comment #8: We have now uploaded high quality figures following the guidelines of PLoS ONE.

On behalf of our co-authors, we hope that our manuscript is now suitable to be considered for review,

Yours sincerely,

Sandrine Gallois and Tinde van Andel
