# Supplementary material for: The importance of choosing appropriate methods for assessing wild food plant knowledge and use: A case study among the Baka in Cameroon
Source: PLoS One. 2021 Feb 18;16(2):e0247108. doi: 10.1371/journal.pone.0247108 (PMC7891729; doi:10.1371/journal.pone.0247108)
Supplement: Attachment — Submitted filename: Response to Reviewer.docx [file pone.0247108.s003.docx]

Anna Fodor	Leiden, 6 January, 2021
Academic Editor
PLOS ONE

Dear Dr. Fodor,

Thank you for allowing us to adapt our manuscript PONE-D-20-37238, entitled “The importance of choosing appropriate methods for assessing wild food plant knowledge and use: a case study among the Baka in Cameroon”. We have followed your suggestions and hope that the manuscript now fits the submission standards of PLoS ONE.

We have submitted a revised version of our manuscript labeled as “revised manuscript with track changes” and a clean copy (unmarked) labeled as “manuscript”, as well as the response to the Editor's comments, Dr Muhammad Ishtiaq, in the document "Response to Reviewer" with a point by point answers.

Below, we list each point made by the academic editor, and our respective answer.

Additional Editor Comments:

Paper needs followings changes/improvements and data provisions; in order to proceed it for further.
**(1)** needs to improve English language, there are many mistakes in the article.(letter of English experts reviewed as supporting needed.

**Answer to comment #1:** Our manuscript was checked by someone with ample experience in publishing scientific articles in the English language.

**(2)** Letter of Ethical and ISE and other institution permission letters are required.

**Answer to comment #2:** The International Society for Ethnobiology does not issue permission letters and does not have an ethnical board: it just published standards for researchers, to which we adhered. However, our research was carried out within the ERC project “HARVEST: Plant Foods in Human Evolution; Factors affecting the harvest of nutrients from the floral environment”. For this research project, our co-author Amanda Henry received approval from the Ethical Board of the Faculty of Medicine of the University of Leipzig, Germany. We have added this letter to our submitted files and listed the HARVEST project in the funding. Moreover, before we started our fieldwork, we received permission from the National Committee of Ethics for the Research of Human Health (CNERSH) of the Cameroonian government. We also added this letter to the submitted files.

**(3)** The references cited in text and reference section needs to be rechecked as it has many mistakes in it.

**Answer to comment #3:** We have checked all references in the text and reference list and adapted them to the style of PLoS ONE.

**(4)** Figure of study area needed.

**Answer to comment #4:** We included a map of the study area (Figure 1)

**(5)** Model of questionnaire needed.

**Answer to comment #5:** We included the model of our questionnaire in our Supporting Information (Supplementary file 1).

**(6)** Families names are not correct, recheck and resend table 1.

**Answer to comment #6:** Family and author names of the species are now listed in the Supporting Information (Supplementary Table 1). All scientific names were checked and updated suing Plants of the World Online.

**(7)** Provide ubiquitous herbarium numbers for all plants collected. it has two types of numbering.

**Answer to comment #7:** Herbarium numbers for all plants collected are listed in Supplementary Table 1, but there are two acronyms (WTH and SG), referring to the two main collectors: Willem Thomas Heger and Sandrine Gallois. We had to follow the Naturalis guidelines of specimen collection for this study.

**(8)** Figures are dim/blurred, send high density/good quality figures.

**Answer to comment #8:** We have now uploaded high quality figures following the guidelines of PLoS ONE.

On behalf of our co-authors, we hope that our manuscript is now suitable to be considered for review,

Yours sincerely,

Sandrine Gallois and Tinde van Andel
