# Supplementary material for: The importance of choosing appropriate methods for assessing wild food plant knowledge and use: A case study among the Baka in Cameroon
Source: PLoS One. 2021 Feb 18;16(2):e0247108. doi: 10.1371/journal.pone.0247108 (PMC7891729; doi:10.1371/journal.pone.0247108)
Supplement: Attachment — Submitted filename: Reviewer Response.docx [file pone.0247108.s005.docx]

**PONE-D-20-37238R1
The importance of choosing appropriate methods for assessing wild food plant knowledge and use: a case study among the Baka in Cameroon.**

**Reviewer Response:**

This manuscript needs following changes/improvements in order to proceed further:

1. In the abstract section, the line number 37 should be rephrased as; We discussed the limitation and strength of these different methods for investigating the diversity of wild food plant; their available knowledge and usage.
2. Grammatical errors should be improved e.g. use of helping verbs, placements of punctuation signs.
   a. Line number 110: Plant knowledge of a certain group of people; should be corrected as; plant knowledge by a certain group of people.
   b. Line number 112: The specimen of species mentioned during free listing should be corrected as; specimen of species identified during free listing.
   c. Line number 115: All useful plants they know and/or use should be corrected as; all useful plants they know or their usage.
3. The line number 38 should be made understandable as; Hence, this analysis will help to further investigate more information regarding wild food plant and their usage frequency, through mixed approach the combines *in-situ and ex-situ methods.*
4. The line number 70; According to Ngome et al.; as reference number is already given, so rearrange the sentence like; According to another study reported,… .
5. From line number 100 to 123; the list of different ethnobotanical method used in this investigation should be included in the methodology section.
6. Line number 156: the sentence should be like; how do different methods for assessing WEP used in this study vary in their results?
7. It is suggested to have a scheme of study i.e. comprehensive framework under which the study is designed and executed e.g. a flow chart or a step by step methodology (figure) followed in this investigation.
8. Line number 180: our inventory of wild food plant knowledge, use and commercialization should be corrected as; our inventory of wild food plant knowledge, their usage and its commercialization.
9. All the tables in the manuscript should be justified as, “Centered- alignment” for all cells of the table; or keep the same alignment for all the tables.
10. Table 1; number of participants should be corrected as; (number of participants= n).
11. Line number 274; kindly mention the figure number.
12. Line number 354; it is recommended to rephrase the heading as; WEP parts used and their preparation method.
13. Line number 336; as this line is a part of table number 2, so try to align it on the same page with the table.
14. Table number 3; please elaborate what denotes “y” here.
15. Line number 525- 541; if this information can be presented in tabular or graphical form/as there is no reference given for any table or figure, that where they are presented.
16. Line number 572; on a population level in our sample should be like; on a population level in our sample collection.
17. Line number 575; kindly correct the formatting mistake.
18. It is suggested that “The accuracy of different methods used in this investigation can be presented either in a graphical or tabular form to highlight their efficiency”.
